# DNA methylation age at birth and childhood: performance of epigenetic clocks and characteristics associated with epigenetic age acceleration in the Project Viva cohort

**DOI:** 10.1186/s13148-023-01480-2

**Published:** 2023-04-12

**Authors:** Anne K. Bozack, Sheryl L. Rifas-Shiman, Diane R. Gold, Zachary M. Laubach, Wei Perng, Marie-France Hivert, Andres Cardenas

**Affiliations:** 1 Department of Epidemiology and Population Health, Stanford University, Research Park, 1701 Page Mill Road, Stanford, CA USA; 2 Division of Chronic Disease Research Across the Lifecourse, Department of Population Medicine, Harvard Medical School and Harvard Pilgrim Health Care Institute, Boston, MA USA; 3 Department of Environmental Health, Harvard T.H. Chan School of Public Health, Boston, MA USA; 4 Channing Division of Network Medicine, Department of Medicine, Harvard Medical School and Brigham and Women’s Hospital, Boston, MA USA; 5 Department of Ecology and Evolutionary Biology (EEB), University of Colorado Boulder, Boulder, CO USA; 6 Department of Epidemiology, Colorado School of Public Health and Lifecourse Epidemiology of Adiposity and Diabetes (LEAD) Center, University of Colorado Anschutz Medical Campus, Aurora, CO USA; 7 Diabetes Unit, Massachusetts General Hospital, Boston, MA USA

**Keywords:** DNA methylation, Epigenetic age, Epigenetic age acceleration, Gestational age acceleration, Epigenetic programming

## Abstract

**Background:**

Epigenetic age acceleration (EAA) and epigenetic gestational age acceleration (EGAA) are biomarkers of physiological development and may be affected by the perinatal environment. The aim of this study was to evaluate performance of epigenetic clocks and to identify biological and sociodemographic correlates of EGAA and EAA at birth and in childhood. In the Project Viva pre-birth cohort, DNA methylation was measured in nucleated cells in cord blood (leukocytes and nucleated red blood cells, N = 485) and leukocytes in early (N = 120, median age = 3.2 years) and mid-childhood (N = 460, median age = 7.7 years). We calculated epigenetic gestational age (EGA; Bohlin and Knight clocks) and epigenetic age (EA; Horvath and skin & blood clocks), and respective measures of EGAA and EAA. We evaluated the performance of clocks relative to chronological age using correlations and median absolute error. We tested for associations of maternal-child characteristics with EGAA and EAA using mutually adjusted linear models controlling for estimated cell type proportions. We also tested associations of Horvath EA at birth with childhood EAA.

**Results:**

Bohlin EGA was strongly correlated with chronological gestational age (Bohlin EGA *r* = 0.82, *p* < 0.001). Horvath and skin & blood EA were weakly correlated with gestational age, but moderately correlated with chronological age in childhood (*r* = 0.45–0.65). Maternal smoking during pregnancy was associated with higher skin & blood EAA at birth [*B* (95% CI) = 1.17 weeks (− 0.09, 2.42)] and in early childhood [0.34 years (0.03, 0.64)]. Female newborns and children had lower Bohlin EGAA [− 0.17 weeks (− 0.30, − 0.04)] and Horvath EAA at birth [*B* (95% CI) = − 2.88 weeks (− 4.41, − 1.35)] and in childhood [early childhood: − 0.3 years (− 0.60, 0.01); mid-childhood: − 0.48 years (− 0.77, − 0.18)] than males. When comparing self-reported Asian, Black, Hispanic, and more than one race or other racial/ethnic groups to White, we identified significant differences in EGAA and EAA at birth and in mid-childhood, but associations varied across clocks. Horvath EA at birth was positively associated with childhood Horvath and skin & blood EAA.

**Conclusions:**

Maternal smoking during pregnancy and child sex were associated with EGAA and EAA at multiple timepoints. Further research may provide insight into the relationship between perinatal factors, pediatric epigenetic aging, and health and development across the lifespan.

**Supplementary Information:**

The online version contains supplementary material available at 10.1186/s13148-023-01480-2.

## Introduction

Human aging is a complex biological process influenced by genetic, environmental, behavioral, and social factors [1]. Blood-based and molecular biomarkers, including inflammatory markers, hormones, telomere length, and epigenetic markers, have been identified to predict age-related outcomes and biological aging [2, 3]. Of particular interest, DNA methylation (DNAm) at specific cytosine-phosphate-guanine (CpG) dinucleotides predictably varies with chronological age and gestational age (GA) [4–6]. Multiple methods have been developed to estimate epigenetic age (EA) in adults and children and epigenetic GA (EGA) in newborns based on DNAm profiles, referred to as epigenetic clocks. Although the development of epigenetic clocks has largely prioritized predicting age-related disease risk and mortality in adults [7], epigenetic clocks in pediatric populations may serve as biomarkers of physiological development or early life programming, and may be responsive to the pre- and perinatal environment.

Epigenetic clocks can be distinguished by the target tissue (e.g., DNAm extracted from saliva or blood), stage of life (e.g., prenatal/birth, childhood/adolescence, or adulthood), and the prediction of age-related traits (e.g., chronological age, mortality, or health span). Epigenetic clocks may be independent of age-related changes in cellular heterogeneity, therefore reflecting intrinsic changes in the epigenome, or may measure extrinsic aging processes dependent on age-related changes in immune cell composition [8]. The weighted average of DNAm at age-related CpGs can be used to estimate EA in a sample. The difference between EA and chronological age, referred to as Epigenetic Age Acceleration (EAA), characterizes an individual’s biological age relative to chronological age. EAA has been shown to be a strong predictor of mortality risk [9–12] and frailty [13], outperforming telomere length [12, 13].

One of the most widely studied epigenetic clocks is the Horvath pan-tissue clock, referred to here as the Horvath clock, which was developed to estimate age across most tissues and cell types [14]. The Horvath clock was trained on tissues and cell types spanning multiple life stages, including cord blood and samples from children and adolescents. Horvath EAA has been associated with physical and cognitive function, cancer, and life expectancy in adults [15, 16], and with development in children and adolescents [17–19]. Horvath et al. additionally developed a clock to estimate age in skin and blood samples, which was trained on DNAm data including cord blood and child buccal cell samples, referred to here as the skin & blood clock [20].

DNAm signatures have also been associated with GA [6, 21]. Epigenetic clocks have been developed to estimate EGA from cord blood [22, 23] or placenta samples [24]. Bohlin et al. leveraged DNAm data measured using the Illumina HumanMethylation450 array (450K) in the Norwegian Mother and Child Birth Cohort study (MoBa) to predict GA, referred to here as the Bohlin clock. Similarly, Knight et al. trained a GA epigenetic clock on DNAm measured using the Illumina HumanMethylation27 (27K) or 450K arrays from six cohorts including multiple ancestries [23], referred to here as the Knight clock. Epigenetic GA Acceleration (EGAA) has been positively associated with birth weight [23, 25, 26], indicating that EGAA is related to developmental maturity, although associations between EGAA and weight may reverse in childhood [26].

**Fig. 1:**
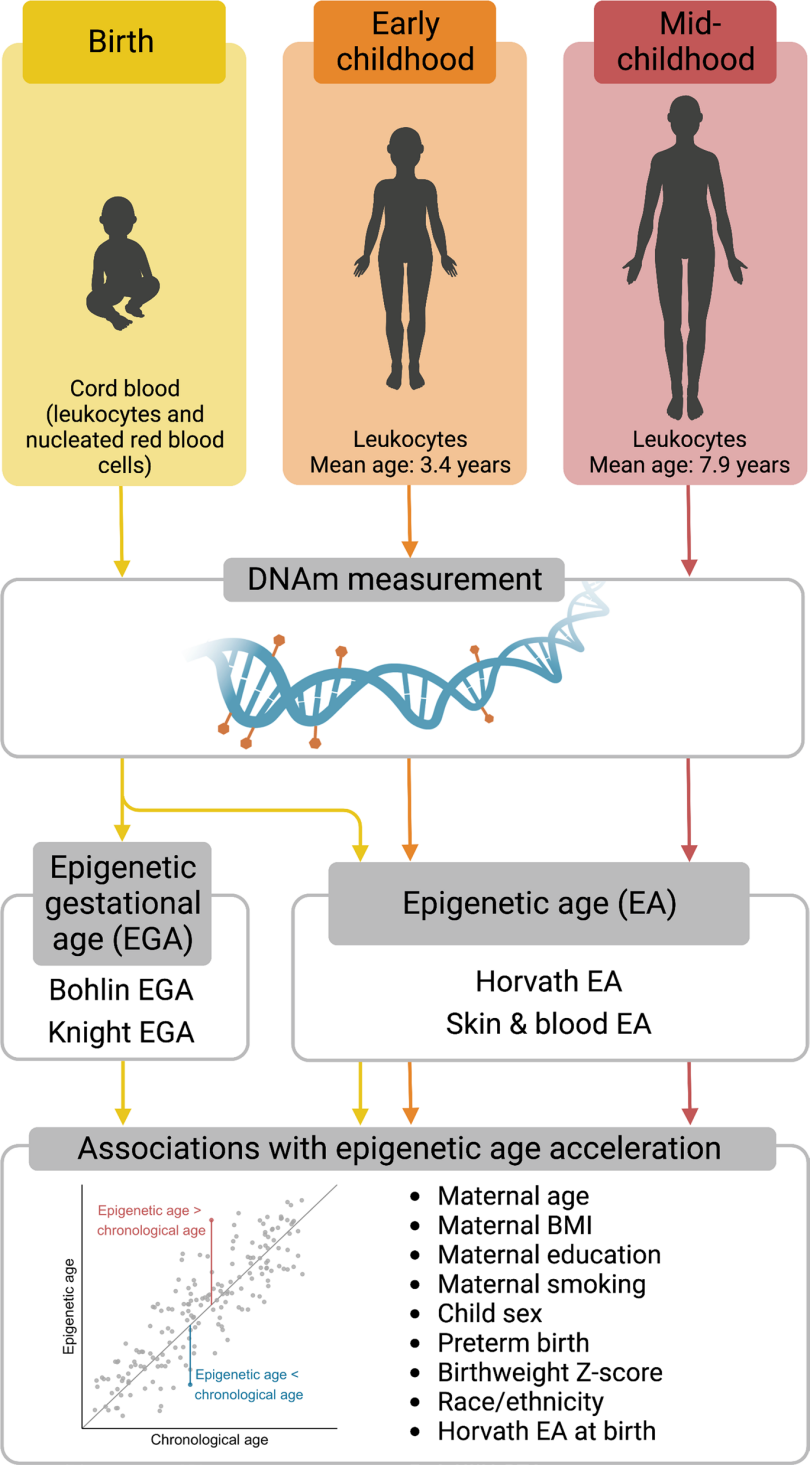
Schematic diagram of study objectives

Increasing evidence suggests that biological factors (e.g., sex and birth weight), conditions affecting the intrauterine environment (gestational diabetes, preeclampsia), social indicators (socioeconomic status and early-life social adversity), and environmental exposures (maternal smoking and air pollution) are associated with epigenetic aging at birth [23, 25–30] and in childhood [31–33]. However, there is limited research on factors that affect EAA at different early-life timepoints, which is important for evaluating the persistence of associations across the lifespan and disease risk. In this study, we examined performance of two clocks developed to estimate EGA (Knight and Bohlin clocks) and two clocks developed to estimate EA across the lifespan (Horvath and skin & blood clocks) in cord blood and blood collected in early and mid-childhood from the Project Viva pre-birth cohort (Fig. 1). We also aimed to identify biological and sociodemographic correlates that may be involved in early life programming and impact epigenetic biomarkers of development. In cross-sectional analyses, we investigated associations between maternal-child characteristics [maternal age, pre-pregnancy body mass index (BMI), education, and prenatal smoking and child sex, preterm birth, birth weight for GA z-score, and self-reported race/ethnicity (a proxy for structural discrimination, racism, and socioeconomic inequality)] and EGAA and EAA at birth and in childhood. We also evaluated associations of Horvath EA at birth with childhood EAA to test the hypothesis that epigenetic age at birth influences biomarkers of development in early life.

## Results

### Study population

Characteristics of mother–child pairs are summarized in Table 1. Data were available for 485 cord blood samples, 120 early childhood blood samples, and 460 mid-childhood blood samples. Mean maternal age at enrollment was approximately 32 years for mother–child pairs with data at birth [mean (standard deviation, SD) = 32.1 (5.3) years], early childhood [mean (SD) = 32.3 (5.0) years], and mid-childhood [mean (SD) = 32.0 (5.6) years]. The majority of mothers had a college or graduate degree (data at birth: 66.4%; early childhood: 71.7%; mid-childhood: 69.5%) and annual household income > $70,000 (data at birth: 60.0%; early childhood: 64.6%; mid-childhood: 64.8%). Approximately 20% of mothers were former smokers (data at birth: 21.0%; early childhood: 23.3%; mid-childhood: 19.1%), and 11–15% reported smoking during pregnancy (data at birth: 11.1%; early childhood: 15.0%; mid-childhood: 11.5%). Approximately half of children were female (data at birth: 47.6%; early childhood: 48.3%; mid-childhood: 47.4%). Child race/ethnicity was reported by mothers. In this analysis, we viewed race and ethnicity as socio-cultural constructs that capture experiences of racism, discrimination, and socioeconomic inequities [34, 35]. Children were classified as Asian (data at birth: 3.3%; early childhood: 5.0%; mid-childhood: 3.0%), Black (data at birth: 12.8%; early childhood: 8.3%; mid-childhood: 19.6%), Hispanic (data at birth: 5.4%; early childhood: 3.3%; mid-childhood: 5.4%), more than one race or other (data at birth: 11.6%; early childhood: 13.3%; mid-childhood: 11.1%), or White (data at birth: 67.0%; early childhood: 70.0%; mid-childhood: 67.8%). For each measure of EA and EAA, means and SDs are included in Table 1. Mean Bohlin EGAA, Knight EGAA, Horvath EAA, and skin & blood EAA was 0.0 for all timepoints.

**Table 1:** Characteristics of mother–child pairs included in analyses of cord blood, early-childhood, and mid-childhood samples

	Cord blood (N = 485)		Early childhood (N = 120)		Mid-childhood (N = 460)	
	n (%)	Missing (n)	n (%)	Missing (n)	n (%)	Missing (n)
Maternal characteristics						
Age at enrollment, years, mean (SD)	32.1 (5.3)	0	32.3 (5.0)	0	32.0 (5.6)	0
Pre-pregnancy BMI, kg/m^2^, mean (SD)	24.8 (5.3)	1	25.9 (6.3)	1	24.9 (5.3)	4
Pre-pregnancy obesity^a^	71 (14.6%)	1	22 (18.3%)	1	70 (15.2%)	4
College graduate	322 (66.4)	0	86 (71.7%)		298 (65.1)	2
Annual household income > $70,000	269 (59.9%)	36	71 (64.6%)	20	254 (60.8%)	42
Smoking status		0		0		0
Former smoker	102 (21.0%)	–	28 (23.3%)	–	88 (19.1%)	–
Smoking during pregnancy	54 (11.1%)	–	18 (15.0%)	–	53 (11.5%)	–
Never smoker	329 (67.8%)	–	74 (61.7%)	–	319 (69.4%)	–
Child characteristics						
Female	231 (47.6%)	0	58 (48.3%)	0	218 (47.4%)	0
Gestational age, weeks, mean (SD)	39.7 (1.6)	0	39.6 (1.6)	0	39.6 (1.6)	0
Preterm^b^	23 (4.7%)	0	7 (5.8%)	0	25 (5.4%)	0
Birth weight for GA z-score, mean (SD)	0.3 (1.0)	0	0.2 (0.9)	0	0.3 (1.0)	1
Race/ethnicity		0		0		1
Asian	16 (3.3%)	–	6 (5.0%)	–	14 (3.0%)	–
Black	62 (12.8%)	–	10 (8.3%)	–	90 (19.6%)	–
Hispanic	26 (5.4%)	–	4 (3.3%)	–	25 (5.4%)	–
More than one race or other	56 (11.6%)	–	16 (13.3%)	–	49 (11.1%)	–
White	325 (67.0%)	–	84 (70.0%)	–	279 (60.7%)	–
Age at sample collection, years, mean (SD)	–	–	3.4 (0.5)	0	7.9 (0.8)	0
Epigenetic age measures						
Bohlin EGA, weeks, mean (SD)	40.3 (1.2)	0	–	–		0
Bohlin EGAA, weeks, mean (SD)	0.0 (0.7)	0	–	–		0
Knight EGA, weeks, mean (SD)	38.8 (1.7)	0	–	–		0
Knight EGAA, weeks, mean (SD)	0.0 (1.4)	0	–	–		0
Horvath EA, years, mean (SD)	0.1 (0.2)	0	4.2 (1.0)	0	9.0 (2.0)	0
Horvath EAA, years, mean (SD)	0.0 (0.2)	0	0.0 (0.8)	0	0.0 (1.8)	0
Skin & blood EA, years, mean (SD)	− 0.3 (0.1)	0	2.6 (0.7)	0	6.5 (1.3)	0
Skin & blood EAA, years, mean (SD)	0.0 (0.1)	0	0.0 (0.5)	0	0.0 (1.1)	0

^a^BMI ≥ 30 kg/m^2^

^b^ < 37 weeks gestation

*EGA*, epigenetic gestational age; *EGAA*, epigenetic age acceleration. *EA*, epigenetic age; *EAA*, epigenetic age acceleration

Data were available at all three timepoints for 59 mother–child pairs, described in Additional File 1: Table S1. There were not significant differences in characteristics of groups with data available at all three timepoints and those with data available at birth (*p* > 0.05, Additional File 1: Table S1). Bohlin EGA was significantly different between newborns with data available at all timepoints and newborns with data available at birth (Mann–Whitney test *p* = 0.020); however, Bohlin EGAA was not significantly different (*p* > 0.05). No other measures of EA or EAA differed significantly between children with data available at all timepoints and children with data available at individual timepoints (Additional File 1: Table S1).

### Performance of epigenetic clocks

**Fig. 2:**
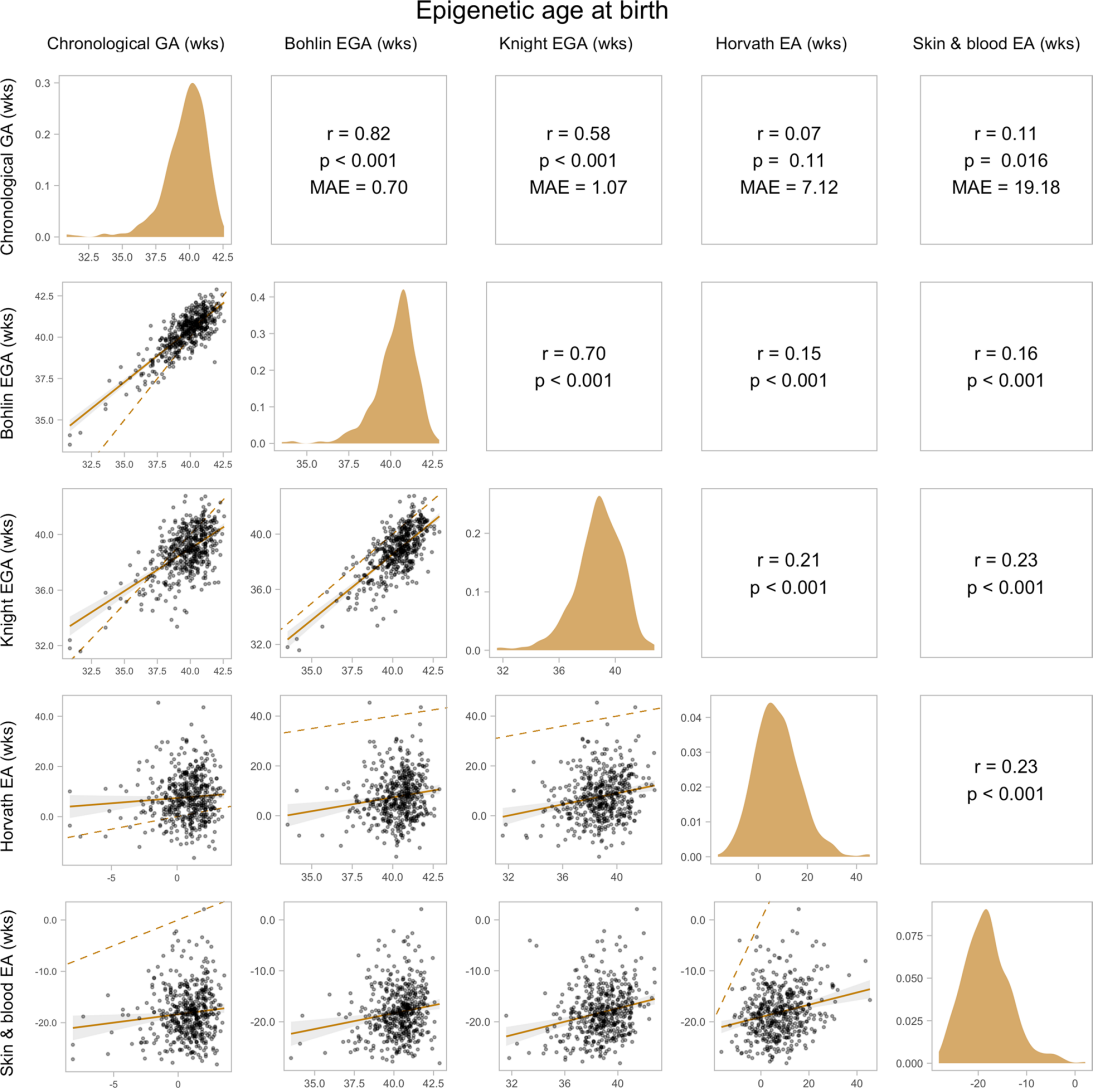
Pairwise relationships between chronological gestational age (GA), epigenetic gestational age (EGA), and epigenetic age (EA) at birth. EGA was estimated using the Bohlin and Knight clocks, and EA was estimated using the Horvath and skin & blood clocks in cord blood (N = 485). The upper panels show the Pearson’s correlation coefficient (*r*), *p*-value (*p*), and median absolute error (MAE) between each pair of variables. The panels on the diagonal show the distributions of each variable. The lower panels show scatter plots of each pair of variables, with the linear trendline and 95% CI plotted as a solid line and shaded area and the identity line plotted as a dashed line

*EGA and EA at birth:* Scatter plots of chronological GA, EGA, and EA (N = 485) are shown in Fig. 2. The Bohlin clock outperformed the Knight clock in accuracy relative to chronological GA as measured by the Pearson correlation coefficient (Bohlin *r* = 0.82; Knight *r* = 0.58; *p* < 0.001) and Median Absolute Error (MAE) (Bohlin MAE = 0.70 weeks; Knight MAE = 1.07 weeks). Skin & blood EA was weakly but significantly correlated with chronological GA (*r* = 0.11; *p* < 0.016). Although Horvath EA was not significantly correlated with chronological GA (*p* > 0.05), it had a smaller MAE than skin & blood EA (Horvath MAE = 7.12 weeks; skin & blood MAE = 19.18 weeks). The majority (80.4%) of estimates for Horvath EA at birth were positive (> 0 years) whereas skin & blood EA was negative for all but one sample.

**Fig. 3:**
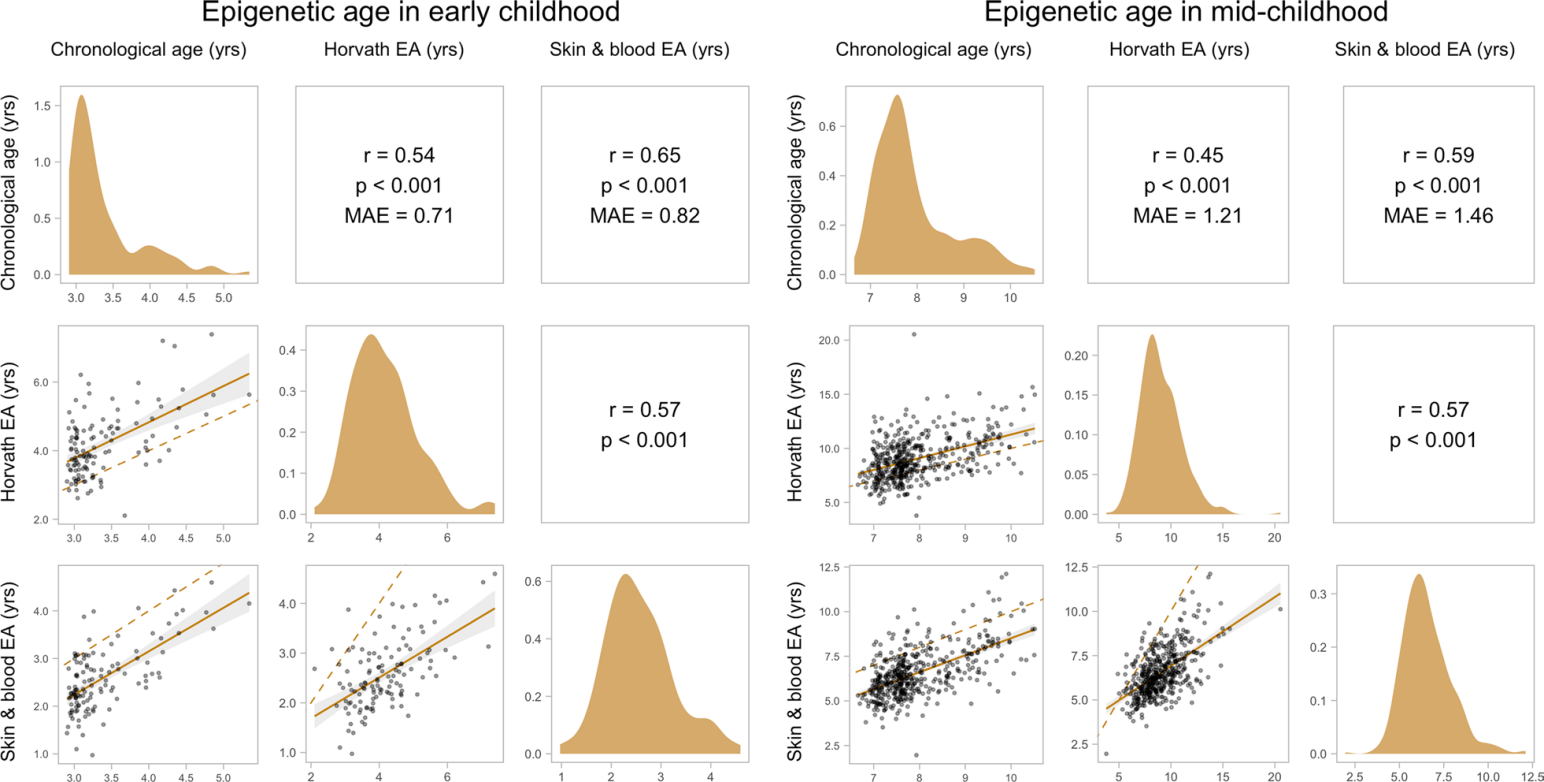
Pairwise relationships between chronological age and epigenetic age (EA) in early mid-childhood. EA was estimated using the Horvath and skin & blood clocks in early (N = 120) and mid-childhood blood (N = 460). The upper panels show the Pearson’s correlation coefficient (*r*), *p*-value (*p*), and median absolute error (MAE) between each pair of variables. The panels on the diagonal show the distributions of each variable. The lower panels show scatter plots of each pair of variables, with the linear trendline and 95% CI plotted as a solid line and shaded area and the identity line plotted as a dashed line

*EA in early and mid-childhood:* Performance of the Horvath and skin & blood clocks in early (N = 120) and mid-childhood (N = 460) is shown in Fig. 3. Horvath EA had a lower MAE in both early (Horvath MAE = 0.71 years; skin & blood MAE = 0.82 years) and mid-childhood (Horvath MAE = 1.21 years; skin & blood MAE = 1.46 years), whereas skin & blood EA had a higher correlation with chronological age in early (Horvath* r* = 0.54; skin & blood *r* = 0.82; *p* < 0.001) and mid-childhood (Horvath *r* = 0.45; skin & blood *r* = 0.59; *p* < 0.001). Both clocks were significantly correlated with each other across timepoints (early childhood: *r* = 0.57; mid-childhood: *r* = 0.57; *p* < 0.001).

We observed that Horvath EA consistently overestimated chronological age in childhood (Fig. 3). After adjusting chronological age for GA (i.e., adding GA in years to chronological age in childhood), the MAE of the Horvath clock significantly decreased from 0.71 to 0.53 years and from 1.21 to 1.03 years in early and mid-childhood, respectively (paired samples Wilcoxon test *p* < 0.001) (Additional File 1: Figure S1).

Both Bohlin and Knight EGA were positively correlated with Horvath and skin & blood EA at birth (N = 485; *p* < 0.001; Fig. 2), and, consequently, Bohlin and Knight EGAA were correlated with Horvath and skin & blood EAA birth (*p* < 0.05; Additional File 1: Table S2). However, Bohlin and Knight EGAA were not significant correlated with Horvath or skin & blood EAA in mid-childhood (N = 238; *p* > 0.05; Additional File 1: Table S2).

### Associations with cell type composition

Although variation in cell type proportions may confound associations between maternal-child characteristics and epigenetic aging, EAA may reflect developmental-related changes in immune cell proportions. We therefore evaluated correlations of cell type proportions estimated from DNAm data with chronological age, EGAA, and EAA, separately.

**Fig. 4:**
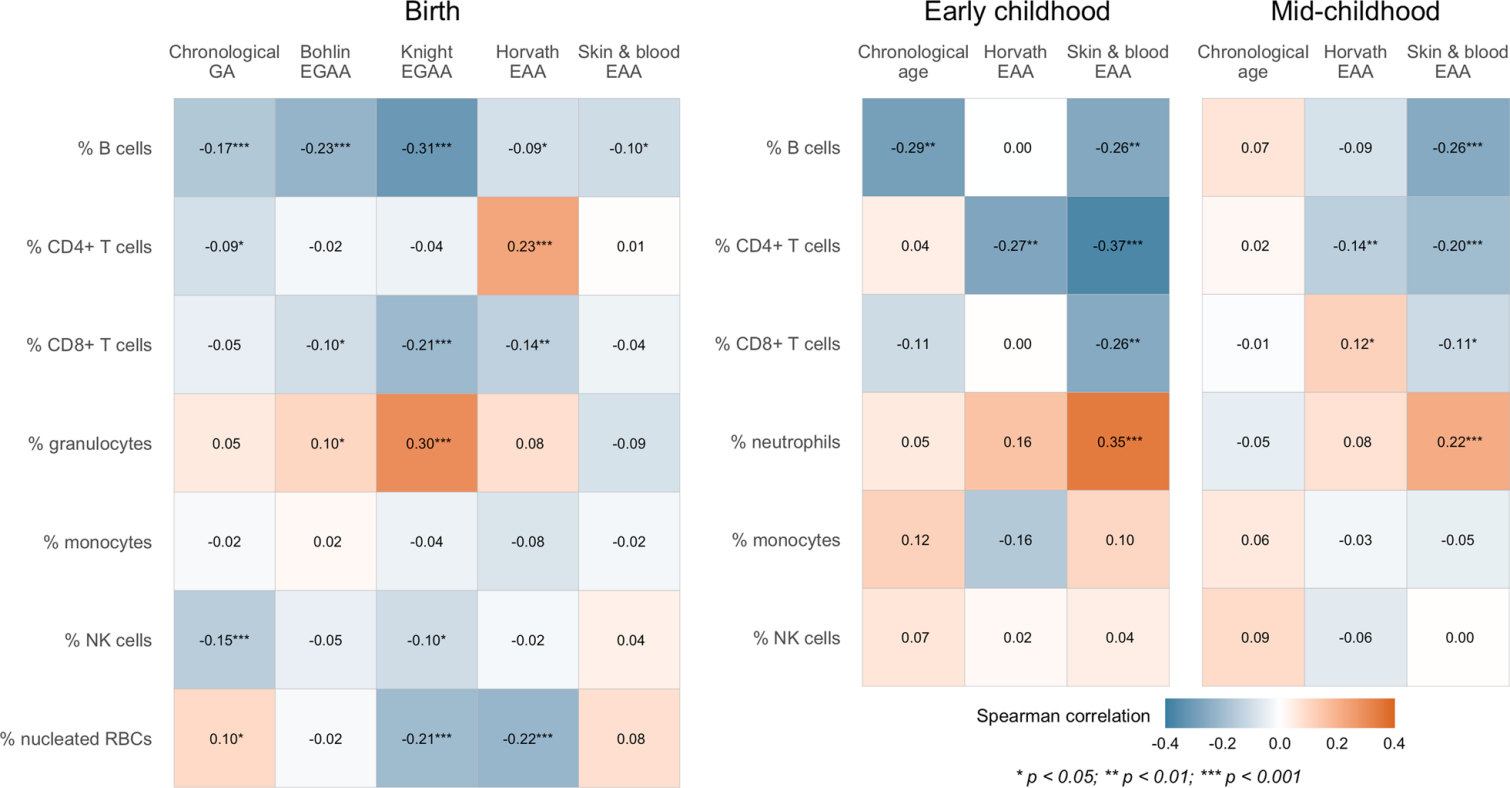
Spearman correlations between cell type proportions estimated from DNAm data, chronological age, and epigenetic age acceleration. GA = gestational age; EGAA = epigenetic gestational age acceleration; EAA = epigenetic age acceleration; NK = natural killer; RBCs = red blood cells

*Associations at birth:* Chronological GA was negatively correlated with % B cells, % CD4+ T cells, and % natural killer (NK) cells, and positively correlated with % nucleated red blood cells (nRBCs) (N = 485; Spearman correlation *p* < 0.05) (Fig. 4). Among the measures of EGAA and EAA at birth, Knight EGAA appeared to be most influenced by cell type, and was negatively correlated with % B cells, % CD8+ T cells, % NK cells, and % nRBCs, but positively correlated with % granulocytes (*p* < 0.05). Correlations between Bohlin EGAA and % B cells, % CD8+ T cells, and % granulocytes were significant and in the same direction, but smaller. Horvath EAA was negatively correlated with % B cells, % CD8+ T cells and % nRBCs and positively correlated with % CD4+ T cells, whereas skin & blood EAA was negatively correlated with % B cells (*p* < 0.05).

*Associations in early and mid-childhood:* Chronological age was negatively correlated with % B cells in early childhood (N = 120; *p* < 0.05); correlations between chronological age and cell type proportions were not significant in mid-childhood (N = 460; Fig. 4). Horvath EAA was negatively correlated with % CD4+ T cells at both childhood timepoints, and positively correlated with % CD8+ T cells in mid-childhood (*p* < 0.05). Skin & blood EAA appeared to better reflect cellular heterogeneity, and was negatively correlated with % B cells, % CD4+ T cells, and % CD8+ T cells, and positively correlated with % neutrophils in early and mid-childhood (*p* < 0.05).

### Associations of maternal-child characteristics with epigenetic age acceleration

**Fig. 5:**
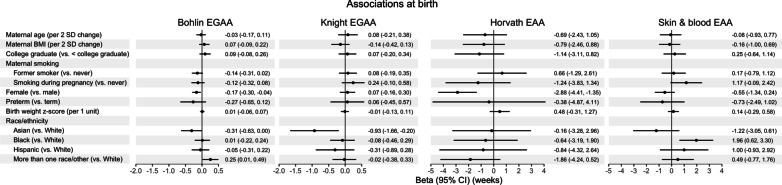
Multivariable associations of maternal-child characteristics with epigenetic gestational age acceleration (EGAA) and epigenetic age acceleration (EAAA) at birth. EGAA was estimated using the Bohlin and Knight clocks, and EAA was estimated using the Horvath and skin & blood clocks in cord blood (N = 484). Associations were evaluated using mutually adjusted robust linear regression controlling for estimated cell type proportions and reported in weeks

*Associations at birth:* Associations of a priori selected variables of maternal age, pre-pregnancy BMI, education, and prenatal smoking and newborn sex, preterm birth, birth weight for GA z-score, and self-reported race/ethnicity with EGAA and EAA at birth were tested using mutually adjusted robust linear models controlling for estimated cell type proportions (N = 484; Fig. 5 and Additional File 1: Table S3). Newborns born to mothers who reported smoking during pregnancy (vs. never smokers) had higher but marginally significant skin & blood EAA [*B* (95% CI) = 1.17 weeks (− 0.09, 2.42)]. Female newborns had lower Bohlin EGAA [*B* (95% confidence interval, CI) = − 0.17 weeks (− 0.30, − 0.04)] and Horvath EAA [*B* (95% CI) = − 2.88 weeks (− 4.41, − 1.35)] compared to male newborns. Asian newborns had lower Knight EGAA [*B* (95% CI) = − 0.93 weeks (− 1.66, − 0.20)]; Black newborns had higher skin & blood EAA [*B* (95% CI) = 1.96 weeks (0.62, 3.30)]; and newborns in the mixed race/ethnicity group had higher Bohlin EGAA [*B* (95% CI) = 0.25 weeks (0.01, 0.49)] compared to White newborns.

Sensitivity analyses were performed without adjusting for cell type proportions to evaluate if observed associations were influenced to variations in immune cell types that may be related to development. Overall, the directions of associations of maternal-child characteristics with EGAA and EAA at birth remained consistent (Additional File 1: Table S3). However, female newborns had higher Knight EGAA than males [*B* (95% CI) = 0.27 weeks (0.03, 0.51)]. In addition, preterm newborns had significantly lower Bohlin [*B* (95% CI) = − 0.42 weeks (− 0.75, − 0.09)], Knight EGAA [*B* (95% CI) = − 0.54 weeks (− 1.02, − 0.07)], and skin & blood EAA [*B* (95% CI) = − 1.94 weeks (− 3.56, − 0.31)].

Results of sex-stratified analyses are in shown Additional File 1: Tables S4 and S5. Maternal pre-pregnancy BMI was associated with higher Bohlin EGAA among female newborns [N = 254; *B* (95% CI) = 0.20 weeks per 2 SD increase in BMI (0.02, 0.37)] but lower Knight EGAA among male newborns [N = 230; *B* (95% CI) = − 0.46 weeks (− 0.83, − 0.09)]. Models including a sex × BMI interaction term suggest significant effect modification for Knight EGAA (Bohlin EGAA *p*_*interaction*_ = 0.46; Knight EGAA *p*_*interaction*_ = 0.024). Birth weight for GA z-score was positively associated with Horvath EAA only among male newborns [*B* (95% CI) = 1.16 weeks per 1 unit increase (0.10, 2.21)], but the interaction term was not statistically significant (*p*_*interaction*_ > 0.05). Sex-specific associations with maternal smoking were also observed. Female newborns born to mothers who were former smokers (vs. never) had lower Bohlin EGAA [*B* (95% CI) = − 0.22 weeks (− 0.44, 0.00)], although there was not a significant sex × smoking interaction (*p*_*interaction*_ > 0.05). Male newborns born to mothers who reported smoking during pregnancy (vs. never) had higher Knight EGAA [*B* (95% CI) = 0.67 weeks (0.24, 1.10)] with a significant sex × smoking interaction (*p*_*interaction*_ = 0.049).

**Fig. 6:**
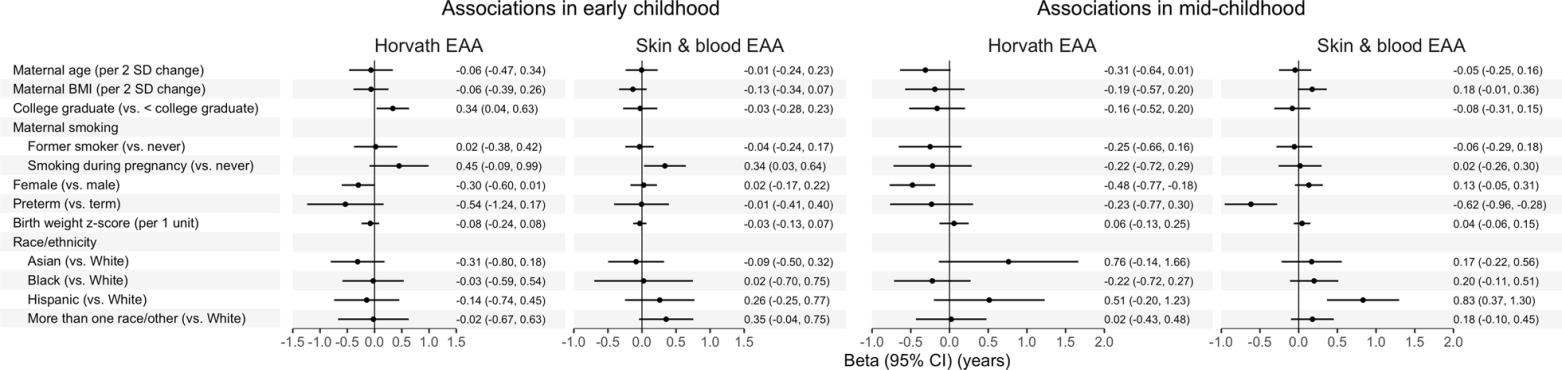
Multivariable associations of maternal-child characteristics with epigenetic age acceleration (EAA) in early and mid-childhood. EAA was estimated using the Horvath and skin & blood clocks in early (N = 119) and mid-childhood blood (N = 455). Associations were evaluated using mutually adjusted robust linear regression controlling for estimated cell type proportions and reported in years

*Associations in early and mid-childhood:* Associations of maternal-child characteristics with EAA in early (N = 119) and mid-childhood (N = 455) from mutually adjusted models controlling for cell type proportions are shown in Fig. 6 and Additional File 1: Table S6. Greater maternal educational attainment (college graduate vs. not) was associated with higher Horvath EAA [*B* (95% CI) = 0.34 years (0.04, 0.63)] and maternal smoking during pregnancy (vs. never) was associated with higher skin & blood EAA [*B* (95% CI) = 0.34 years (0.03, 0.43)] in early childhood. Children’s characteristics (sex, preterm birth, birth weight for GA z-score, and race/ethnicity) were not significantly associated with EAA in early childhood (*p* > 0.05). In mid-childhood, females had lower Horvath EAA [*B* (95% CI) = − 0.48 years (− 0.77, − 0.18)] and children who were born preterm had lower skin & blood EAA [*B* (95% CI) = − 0.62 years (− 0.96, − 0.28)]. In addition, Hispanic children had higher skin & blood EAA compared to White children in mid-childhood [*B* (95% CI) = 0.83 years (0.37, 1.30)].

Without cell type adjustment, associations of maternal-child characteristics with EAA in childhood remained consistent overall (Additional File 1: Table S6). Associations of maternal education with Horvath EAA [*B* (95% CI) = 0.31 years (− 0.03, 0.65)] and maternal smoking during pregnancy with skin & blood EAA [*B* (95% CI) = 0.26 years (− 0.01, 0.54)] in early childhood were attenuated.

In sex-stratified analyses, maternal smoking during pregnancy (vs. never) was associated with significantly higher early childhood skin & blood EAA only among female children [N = 58; *B* (95% CI) = 0.53 years (0.17, 0.88)] (Additional File 1: Tables S7 and S8), although there was not a significant sex × smoking interaction (*p*_*interaction*_ > 0.05). In addition, preterm birth was associated lower Horvath EAA among female children in early childhood [*B* (95% CI) = − 0.59 years (− 1.14, − 0.04)] and among male children in mid-childhood [N = 237; *B* (95% CI) = − 0.63 (− 1.25, − 0.01)]. Interaction terms were not significant at either timepoint (*p*_*interaction*_ > 0.05).

### Horvath epigenetic age at birth is associated with epigenetic age acceleration in childhood

**Fig. 7:**
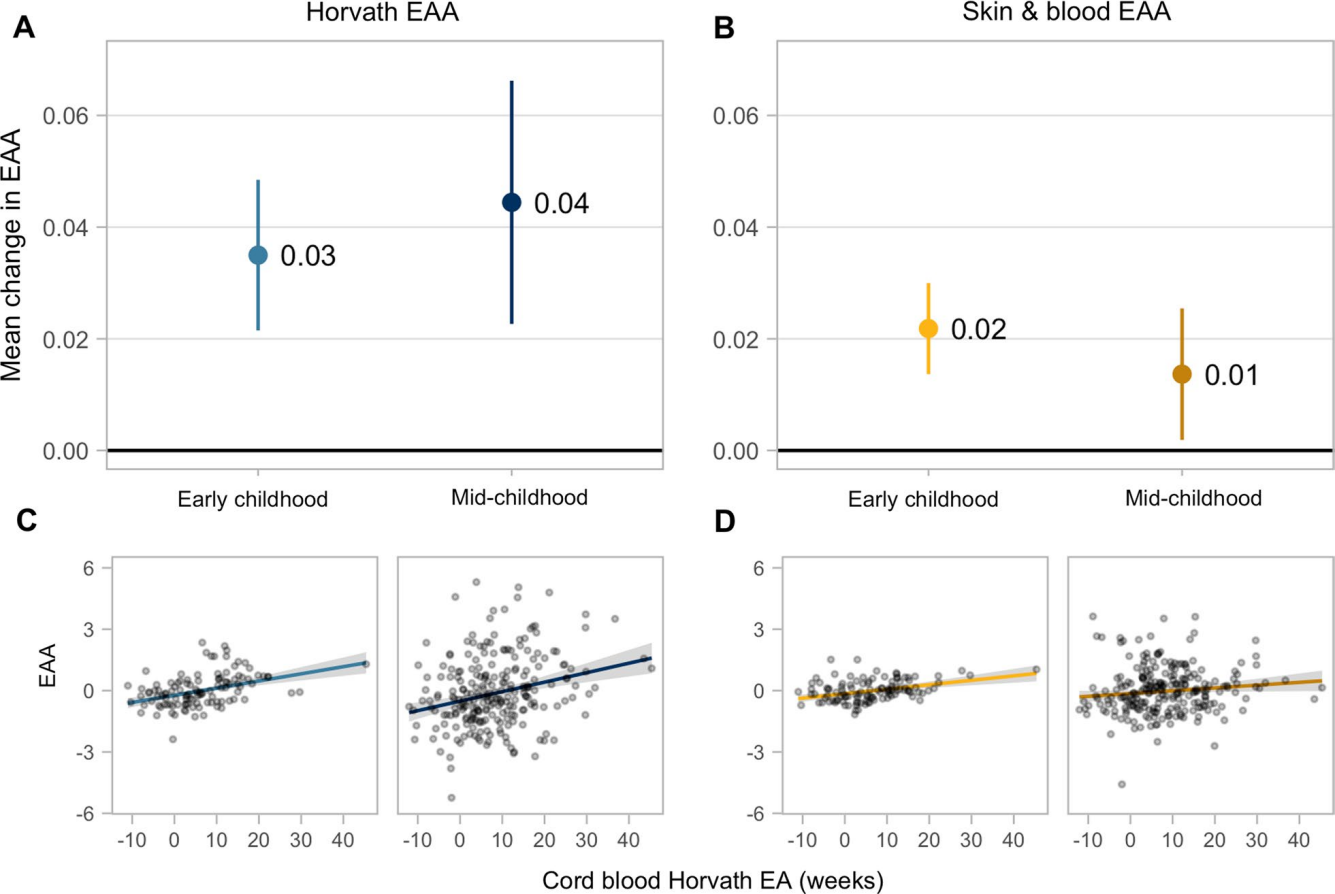
Associations between Horvath epigenetic age (EA) at birth and epigenetic age acceleration (EAA) in early and mid-childhood. Horvath EA was estimated using the Horvath clock in cord blood, and Horvath EAA was estimated using the Horvath clock in early (N = 113) and mid-childhood blood (N = 238). Effect estimates and 95% Cis from robust linear regression evaluating associations between Horvath EA in cord blood and Horvath and skin & blood EAA shown in the top left and right panels (**A**–**B**), respectively. Scatter plots of Horvath EA in cord blood and Horvath and skin & blood EAA shown in the bottom left and right panels, respectively (**C**–**D**)

Observing that Horvath EA was significantly associated with the other clocks at birth, although not with chronological GA, we hypothesized that Horvath EA captures aspects of biological age independent of chronological age. Horvath EA and EAA in at birth were highly correlated (N = 485; *r*_*Pearson*_ = 0.99; *p* < 0.001) due to the narrow range of chronological GA; therefore, we evaluated associations of Horvath EA at birth with childhood EAA. In unadjusted robust linear models, higher Horvath EA at birth was consistently associated with higher Horvath EAA and skin & blood EAA in early (N = 113) and mid-childhood (N = 238) (Fig. 7 and Additional File 1: Table S9). Associations between Horvath EA at birth and Horvath EAA were similar in early [*B* (95% CI) = 0.03 years per week of Horvath EA at birth (0.02, 0.05)] and mid-childhood [*B* (95% CI) = 0.04 years (0.02, 0.07)]. On average, compared to a child at the 25th percentile of Horvath EA at birth, a child at the 75th percentile had 0.44 years higher EAA in early childhood and 0.54 years higher EAA in mid-childhood. Associations between Horvath EA at birth and skin & blood EAA in childhood were positive but had a smaller effect size [early childhood* B* (95% CI) = 0.02 years (0.01, 0.03); mid-childhood *B* (95% CI) = 0.01 years (0.00, 0.03)]. In fully adjusted models, we observed similar effect sizes (early childhood: N = 112; mid-childhood: N = 238; Additional File 1: Table S9).

## Discussion

This study aimed to evaluate the performance and identify biological and sociodemographic correlates of two EGA clocks and two pan- or multi-tissue EA clocks at birth and during follow-up at two timepoints in childhood in the Project Viva pre-birth cohort. Estimates of Horvath and skin & blood EA were close to 0 years at birth, but weakly correlated with chronological GA. However, Horvath and skin & blood EA were moderately correlated with chronological age in early and mid-childhood. We observed significant associations between maternal-child characteristics and EAA, although relationships varied across clocks and ages. Maternal smoking during pregnancy was associated with higher skin & blood EAA at birth and in early childhood. Child sex was most consistently associated with differential EGAA (female newborns having lower Bohlin EGAA) and EAA (female newborns and children having lower Horvath EAA). Associations of child race/ethnicity with EAA were also identified across clocks.

Bohlin and Knight EGA were significantly correlated with chronological GA at birth; however, Bohlin EGA had notably higher correlation (N = 485; Bohlin *r*_*Pearson*_ = 0.82 vs. Knight *r*_*Pearson*_ = 0.58) and lower MAE (Bohlin MAE = 0.70 vs. Knight MAE = 1.07 weeks) relative to chronological GA. This observation is consistent with previous studies demonstrating better prediction of GA at birth using Bohlin EGA compared to Knight EGA [25–27, 29, 36]. Several factors in the development of the Knight clock may contribute to its lower performance, including training on GA determined by a combination of LMP, ultrasound, and clinician report [23] and potential overfitting due to the high ratio of CpGs included in this clock to training samples [37]. It should also be noted that although the development of the Knight clock included newborns of multiple ancestries, the training datasets included in both the Bohlin and Knight clocks were from predominantly White populations [22, 23], which may have affected performance among newborns in other race/ethnicity groups in our study.

Although the Horvath and skin & blood clocks were developed to estimate chronological age in a range of tissue types across the life course and included cord blood in training sets, these clocks were not optimized to estimate GA. For example, the training age for cord blood samples was set to 0 years for the Horvath clock [14]. However, it has previously been demonstrated that these clocks are correlated with GA in cord blood and blood spot samples (*r* = 0.15–0.65 for the Horvath clock and *r* = 0.18–67 for the skin & blood clock) [20]. In our study, the Horvath and skin & blood EA were positively but weakly correlated with GA. Performance of both clocks was similar at early- and mid-childhood, although Horvath EA had a lower MAE and skin & blood EA had a higher correlation with chronological age.

Children born prematurely (< 37 weeks gestation) had significantly lower EAA in childhood. Preterm birth was associated with lower skin & blood EAA among children overall in mid-childhood [N = 455; *B* (95% CI) = − 0.62 years (− 0.96, − 0.28)], Horvath EAA among female children in early childhood [N = 58; *B* (95% CI) = − 0.59 years (− 1.14, − 0.04)], and Horvath EAA among male children in mid-childhood [N = 237; *B* (95% CI) = − 0.63 (− 1.25, − 0.01)]. In agreement with a previous study of EGAA [27], we also found that preterm birth was negatively associated with EGAA and EAA at birth without adjusting for estimated cell type proportions. Preterm birth has previous been associated with lower height and weight in childhood, adolescence, and early adulthood [38–41], which may be reflected in epigenetic age biomarkers. In addition, greater variation in height, weight, and head circumference has been found among male children born prematurely compared to female children [39]. Although sex × preterm birth interaction terms were not statistically significant in our study, larger sample sizes are needed to determine if sex modifies the effect of preterm birth on EAA in childhood.

We found evidence of associations between maternal smoking during pregnancy and higher skin & blood EAA at birth [N = 484; *B* (95% CI) = 1.17 weeks (− 0.09, 2.42) for smoking during pregnancy vs. never] and early-childhood [N = 119; *B* (95% CI) = 0.34 years (0.03, 0.64)]. Prenatal maternal smoking has previously been associated with accelerated skin & blood aging in children 6–11 years old in the Human Early-Life Exposome project, which found a similar effect size [*B* (95% CI) = 0.15 years for maternal prenatal smoking vs. not (0.02, 0.28)] [31]. Other studies have reported that maternal smoking during pregnancy increases EGAA in placenta [28], cord blood, and chorionic villus samples [29]; although, concordant with our results, null results have also been reported with Bohlin and Knight EGAA [27].

We observed evidence of sex-specific effects of maternal pre-pregnancy BMI and smoking during pregnancy on EGAA, as well as significant associations of child sex with multiple EAA biomarkers. For measures of EGAA, females had lower Bohlin EGAA in models adjusted for cell type [N = 484; *B* (95% CI) = − 0.17 weeks (− 0.30, − 0.04)] but higher Knight EGAA in models without cell type adjustment [*B* (95% confidence interval [CI]) = 0.27 weeks (0.03, 0.51)]. These effect estimates are small and the discrepancy in results may be due in part to the treatment of sex in the development of EGA clocks. Knight et al. did not include sex as a covariate but reported EGA to be independent of sex [23], and Bohlin et al. adjusted for sex in EWAS to select CpGs for inclusion in elastic net prediction models [22]. Our results may also indicate that sex-related Knight EGAA is influenced by extrinsic processes (i.e., dependent on developmental-related changes in immune cells) whereas Bohlin EGAA is more reflective of intrinsic processes (i.e., independent of cell type proportions). Female sex has been previously associated with higher Knight EGAA with similar effect sizes in adjusted analyses of two separate birth cohorts as reported by Daredia et al. [*B* (95% CI) = 0.45 weeks (0.15, 0.75)] [27] and Girchenko et al. [*B* (95% CI) = 0.30 weeks (0.05, 0.55) for EGAA calculated as epigenetic GA – chronological GA] [30]. Also consistent with our analyses, Khouja et al. reported lower Bohlin EGAA among female newborns [25]. Additionally, among females, we found lower Horvath EAA at birth [N = 484; *B* (95% CI) = − 2.88 weeks (− 4.41, − 1.35)] and in mid-childhood [N = 455; *B* (95% CI) = − 0.48 years (− 0.77, − 0.18)]. A previous study found no sex differences in Horvath EAA in children calculated from buccal DNAm [42]. However, analyses of cord and peripheral blood in the Avon Longitudinal Study of Parents and Children (ALSPAC) cohort identified a negative correlation between female sex and Horvath EAA, which increased with age [43]. Similarly, in cord blood samples from the GOYA study, females had lower Horvath EAA at birth [*B* (95% CI) = − 3.12 weeks (− 4.68, − 1.56)] [43]. Lower Horvath EAA among females has also been found in adult populations [8], consistent with our findings.

Our study identified associations of child race/ethnicity with EGAA and EAA. Children’s race/ethnicity was reported by mothers and classified based on commonly used categories in epidemiology, which reflect aspects of cultural and social factors, racism, discrimination, and socioeconomic inequities [34]. In adults, racial and ethnic differences in EAA have also been observed, although associations differed in significance and direction between intrinsic and extrinsic measures [8]. As a socially defined construct, self-reported race/ethnicity is most likely a risk marker, rather than a risk factor [34], of differential EAA. Associations observed in our study may reflect upstream determinants of health and child development, including socioeconomic inequities and maternal or child experiences of structural racism and discrimination. Although we found limited evidence of associations of maternal age and education with EAA at birth or in childhood, prenatal socioeconomic status has been associated with DNAm levels at individual loci in Project Viva [44], and previous studies have reported accelerated epigenetic aging among adults with lower socioeconomic indicators [45, 46], neighborhood deprivation [47], and social class in childhood [48, 49]. It should also be noted that interpretation of associations with race/ethnicity is limited in this study due to a population with relatively high socioeconomic status (i.e., all mother–child pairs had health insurance), which may not be generalizable to other populations; a small number of children in the Hispanic and Asian categories, increasing the risk of bias; and inclusion of a “more than one race or other” category, which may mask variation within this group.

The clocks included in this study may capture aspects of both intrinsic and extrinsic developmental and aging processes. Intrinsic age is understood as being independent of variation in cell type composition, particularly cellular subtypes known to change with age, whereas extrinsic age reflects processes related to the immune system and blood cell heterogeneity [50]. We observed correlations between estimated cell type proportions and all EAA biomarkers, although associations were not consistent across clocks or timepoints. Although both Bohlin et al. and Knight et al. reported negligible effects on the performance of GA clocks with adjustment for cell type [22, 23], in our study, Knight EGAA was most strongly correlated with cell type and therefore may indeed capture immune cell changes related to fetal development. In addition, we found associations between cell type proportions and Horvath EAA, a biomarker that performs well across tissue types and is considered to primarily measure intrinsic aging. As suggested by Horvath, our observed associations may be due to confounding by cell types that vary with age [14].

Overall, Horvath EA overestimated chronological GA and age in early and mid-childhood. Adding GA to childhood chronological age significantly reduced the MAE, suggesting that the Horvath clock begins “ticking” at conception rather than at birth. Although the Horvath clock is trained on a transformed version of chronological age to account for more rapid changes in DNAm early in life [51] and is a robust tool to estimate age across the life course, it may be less precise in newborn and pediatric populations [52]. However, our observation also suggests that the Horvath clock may be capturing intrinsic age-related changes to the methylome that begin with epigenetic reprogramming during embryogenesis. In fact, Ingenuity Pathway Analysis (IPA) of the CpGs included in the Horvath clock indicated enrichment for cellular growth and proliferation and organism, embryo, and tissue development [14]. We also identified Horvath EA at birth as the most significant variable associated with Horvath EAA in childhood, suggesting that EA at birth strongly influences the rate of epigenetic aging or development in early life. This apparent programming of epigenetic aging may be due in part to genetic factors, which have been shown to influence age-related changes in the methylome [53], while other factors (e.g., environmental exposures, lifestyle factors, epigenetic drift) may have an increasing effect on EAA over the life course. Horvath investigated heritability of aging in twin datasets from newborns and adults, finding 100% heritability of EAA at birth measured by Falconer’s formula in contrast to 39% heritability in older adults [14]. In our study, although associations were weaker, Horvath EA at birth was also significantly associated with skin & blood EAA in early and mid-childhood, despite minimal overlap in CpGs used to predict age between the clocks. These results provide evidence that common pleiotropic genetic variants are related to these biomarkers. A GWAS of intrinsic Horvath EAA found minimal co-localization of aging-related SNPs and CpGs used to derive Horvath EA [54].

In adult populations, higher EAA is a predictor of a broad spectrum of aging-related outcomes including frailty, cancer incidence, cancer and cardiovascular mortality, and all-cause mortality [9, 13, 16, 55–58]; whereas lower EA may be associated with longevity [59]. Our study focused on first-generation epigenetic clocks, which have been trained to predict chronological age. Second-generation clocks, i.e., clocks trained to predict aging-related physiological outcomes or risk scores, such as PhenoAge [56] and GrimAge [60], outperform first-generation clocks as predictors of mortality, health span, and morbidity [15, 56, 61, 62], However, these clocks were trained on adult samples [56, 60] and their training phenotypes may be less relevant for pediatric populations. There is some evidence that early life EAA from first-generation clocks is positively associated with developmental markers, which may have health implications later in life. In ALSPAC, Horvath EAA at birth was positively associated with fat mass throughout childhood and adolescence [*B* (95% CI) = 1321 g per year of EAA (386, 2256)] and EAA at 7 years was positively associated with height [*B* (95% CI) = 0.23 cm per year of EA (0.04, 0.41)] [17]. In adolescents, greater Horvath EAA has also been associated with earlier pubertal development [18, 19]. Epigenetic clocks developed to predict age across the lifespan and those developed to predict GA may be distinct biomarkers relative to fetal development and developmental trajectories. Although EGAA has also been positively associated with measures of developmental maturity in newborns, including birth weight, length, and head circumference [25, 26, 29], the association between EGAA and development may reverse in childhood, as suggested by a longitudinal analysis in ALSPAC finding a negative association between EGAA and weight after age 5 [26]. Other studies, however, have found a null [27] or inverse association [30] between EGAA and developmental indices at birth, although comparison across studies may be impacted by methodological differences including adjustment for cell type proportions and use of raw differences, rather than residuals, to calculate EGAA. These results highlight that study of the early life EAA/EGAA and development is a relatively nascent area of research, and further studies are needed to fully understand the relationship between epigenetic clocks and health in pediatric populations.

Strengths of this study include our ability to leverage DNAm data collected at multiple ages from a well-established pre-birth cohort. Cord blood and blood collected at early and mid-childhood represent important developmental stages that may be differentially responsive to prenatal biological, socioeconomic, and environmental factors. To our knowledge, few studies have examined EAA across multiple early-life stages [36]. Although our analyses were cross-sectional and a limited number of mother–child pairs had data at all three timepoints (N = 59), there were not significant differences between mother–child pairs with data at all three timepoints and pairs with data at birth. Our study was also strengthened by calculating EA using multiple clocks developed to estimate GA in cord blood (Bohlin and Knight clocks) and chronological age across the lifespan (Horvath and skin & blood clocks), which allowed us to compare characteristics associated with early life epigenetic aging across multiple biomarkers.

Our study had several limitations. Chronological GA was calculated using LMP or ultrasound if data were available and if ultrasound estimates differed from LMP GA estimates by > 10 days. Although we do not expect this method to introduce systematic bias, it may decrease precision relative to the gold standard of GA determined by ultrasound [63]. DNAm was not measured in all Project Viva participants and was restricted to participants with proper consent for genetic and epigenetic analyses and available samples with sufficient quantity. Although this limited our samples size, we do not believe that available data would bias results. Our relatively small sample size reduced our power to detect small effect sizes and sex-specific effects. Given the discovery nature of this study, we chose not to adjust for multiple comparisons (i.e., multiple EGAA or EAA outcomes) to allow us to identify associations that may be validated in larger studies. Analyses of early and mid-childhood EAA were limited to associations with prenatal factors; environmental exposures and socioeconomic status during childhood may act to amplify or reverse the effects of prenatal factors, a topic that should be further explored. Furthermore, our interpretation of findings was impacted by the limitations of available epigenetic clocks. Although the Bohlin, Knight, Horvath, and skin & blood clocks are commonly used in epidemiological studies including ethnically diverse and pediatric populations [52], racial/ethnic differences in epigenetic aging are not fully understood [8], and these clocks may introduce bias when applied to multi-ethnic cohorts. In addition, few clocks have been developed specifically for the pediatric population [52], and existing clocks are trained on chronological age, making it difficult to interpret the biological or clinical significance of variation in EAA. Integrating age with age and disease-related biomarkers has produced clocks that are highly predictive of health outcomes in adults [15]; similar approaches may advance the development of epigenetic biomarkers of development in the pediatric population.

## Conclusion

This study provides evidence that sex, self-reported race/ethnicity, and factors impacting the intrauterine environment, particularly maternal smoking during pregnancy, may be associated with EAA at birth and in childhood. However, the limited consistency of findings across biomarkers suggests that each clock captures unique aspects of biological development and aging. By examining the Horvath clock at multiple timepoints, we observed that this clock may start “ticking” prior to birth, and that that Horvath EA at birth is strongly associated with EAA in childhood, which may be driven by genetic factors and fetal programming. Further research is needed to fully understand the complex relationship between the biological and sociodemographic correlates of these epigenetic biomarkers and health across the lifespan, as well as to develop pediatric epigenetic clocks suitable for diverse populations.

## Methods

### Study design

We leveraged data from Project Viva, a longitudinal pre-birth cohort designed to examine associations between maternal diet, environmental factors, and maternal and child health [64]. In brief, between 1999 and 2002, we recruited pregnant women from Atrius Harvard Vanguard Medical Associates, a group practice in Eastern Massachusetts, US. At their initial obstetric visit (median gestation: 9.9 weeks), participants were screened by research staff. Participants were excluded if they had a multiple gestation, were ≥ 22 weeks gestation, were not English speaking, or planned to move out of the study area prior to delivery. A total of 2670 pregnancies (64% of those screened) were enrolled, and 2128 live births remained in the cohort at the time of delivery.

Study visits were conducted by research assistants at enrollment in early pregnancy, mid-pregnancy, hospital birth admission, infancy, and early and mid-childhood (median child age = 3.2 years and 7.7 years, respectively). All mothers provided written informed consent at enrollment and at childhood visits. Biospecimen collection protocols were designed to minimize discomfort and inconvenience for children and mothers. All study protocols were approved by the Institutional Review Board (IRB) at Harvard Pilgrim Health Care (IRB reference # 235,301).

### Biospecimen sample collection, processing, and DNA methylation analysis

At delivery, clinical staff were prompted to collect cord blood samples using paper or electronic “flags” on mothers’ charts. Clinicians collected cord blood from the umbilical vein using a syringe and needle. Blood samples were collected from children at the early and mid-childhood visits. Within 24 h of collection, cord blood and childhood blood samples were centrifuged at 1700 × g for 10 min at 4 °C to separate plasma, red blood cells (RBCs), and nucleated cells used for measurement of DNAm (leukocytes and nRBCs in cord blood, and leukocytes in childhood blood).

DNAm was measured in all participants with proper consent for genetic and epigenetic analyses and sufficient sample quantity. Quantification of DNAm has been detailed previously [65]. Research staff extracted genomic DNA from nucleated cells using the PureGene kit (Qiagen, Valencia, CA) and stored sample aliquots at – 80 °C until analysis. DNA was bisulfite converted with the Zymo DNA Methylation kit (Zymo Resarch, Irvine, CA). DNAm was analyzed at Illumina, Inc. with the Illumina 450K Beadchip (Illumina, San Diego, CA), which interrogates > 485,000 methylation sites. To minimize batch effects, 1 μg of DNA from each sample was randomized across plates and BeadChips.

### Data processing

We processed DNAm data using the R package *minfi* [66]. We excluded samples based on the following criteria: duplicate samples, low individual call rates (< 0.98), and genotyping or sex mismatch. Background correction and dye-bias equalization was performed using the normal-exponential out-of-band method (noob) [67], and probe-type normalization was performed using the beta-mixture quantile method (BMIQ) [68]. *Combat* in the *sva* package [69] was applied to adjust for variability associated with plate and scanner. We estimated cell type proportions using Houseman’s method based on regression calibration [70] implemented in *minfi* with reference panels derived from nucleated cord blood cells [71] (to estimate B cell, CD4+ T cell, CB8+ T cell, granulocyte, monocyte, NK cell, and nRBC proportions) or adult leukocytes [72] (to estimate B cell, CD4+ T cell, CB8+ T cell, neutrophil, monocyte, and NK cell proportions).

### Covariates

We collected maternal sociodemographics, smoking habits before and during pregnancy, and self-reported pre-pregnancy weight and height using self-administered questionnaires and interviews. We asked mothers to report their child’s race/ethnicity, and classified responses as “White”, “Black”, “Hispanic”, “Asian”, “more than one race”, and “other”. For the current analyses, “more than one race” and “other” were combined due to low sample sizes in these categories. Pre-pregnancy BMI in kg/m^2^ was calculated from self-reported weight and height. We estimated chronological GA using mothers’ last menstrual period (LMP) reported at enrollment. If GA assessed by ultrasound was available and differed from LMP estimates by more than 10 days, the value derived from ultrasound was used [64]. We calculated sex-specific birth weight for GA z-scores using a US national reference [73].

### Data analysis

For cord blood samples, we estimated Bohlin EGA using the *Gaprediction* package [22], which is based on a model trained on GA estimated with ultrasound. We set the Lasso penalty parameter to be the minimum lambda, which uses 251 CpGs, as this method minimized the MAE between estimated and chronological GA in our data. We also estimated EGA using Knight et al.’s method implemented with normalization using R code provided with the publication [23]. For both clocks, EGAA was calculated as the residuals of regressing EGA on chronological GA. For cord blood and early and mid-childhood blood samples, we calculated EA with the Horvath clock [14] and the skin & blood clock [20] using Horvath’s new online calculator with normalization (http://dnamage.genetics.ucla.edu/). The online calculator also provides calculations of EAA using the residual method for each clock. A summary of the clocks used in these analyses, including the training sets and number of CpGs, is included in Additional File 1: Table S10. The number of CpGs common overlapping between the clocks were: Horvath and skin & blood: 60 CpGs; Knight and Horvath: 6; Bohlin and Knight: 2; Bohlin and skin & blood: 1 (Additional File 1: Figure S2).

We evaluated the performance of each DNAm clock by calculating Pearson correlations and MAE between estimated and chronological age. To evaluate performance of the Horvath clock and skin & blood clock at birth, we converted GA in weeks to years [(gestational week – 39)/52], as previously applied by Horvath et al. [20]. We also calculated Spearman correlations between EGAA and EAA at birth and mid-childhood using Spearman correlations. Descriptive statistics of covariates were calculated as the mean and SD for continuous variables, and frequency and proportion for categorical variables. We evaluated differences between mother–child pairs with data at all three timepoints (birth, early childhood, and mid-childhood, N = 59) and pairs with data at birth (N = 485) using the Mann–Whitney test for continuous variables and the Chi-squared test or Fisher’s exact test for categorical variables.

Spearman correlations were calculated between estimated blood cell type proportions and chronological age, EGAA, and EAA. Associations of maternal-child characteristics and EGAA or EAA were analyzed using robust linear models to reduce the influence of extreme values. Models were evaluated using the *rlm* function in the *MASS* package [74] in R with the M estimator. We calculated *p*-values and 95% CIs using the *lmtest* package [75] implemented with the vcovHC covariance matrix estimation function with White’s estimator [76] from the *sandwich* package [77, 78]. In cord blood samples, associations with Bohlin EGAA, Knight EGAA, Horvath EAA, and skin & blood EAA were tested; in early and mid-childhood blood samples, associations with Horvath EAA and skin & blood EAA were tested. The following maternal-child characteristics were selected a priori and evaluated as independent variables in mutually adjusted models: maternal age at enrollment, pre-pregnancy BMI, education (college graduate vs. not), and self-reported maternal smoking (former smoker or smoking during pregnancy vs. never smoker) and child sex, preterm birth, birth weight for GA z-score, and self-reported race/ethnicity (Black, Hispanic, Asian, more than one race/other vs. White). For interpretation of effect estimates, maternal age and pre-pregnancy BMI were mean centered and scaled by dividing by 2 × the standard deviation [79]. Variation in cell type proportions may be a confounder of the association between maternal-child characteristics and EAA, or EAA may capture age-related changes in immune cell proportions. Therefore, models were evaluated with and without including estimated blood cell type proportions as potential confounders. We evaluated effect modification by child sex using stratified analyses and interaction terms in mutually adjusted models including cell type estimates. In addition, associations between Horvath EA at birth and EAA in early and mid-childhood were evaluated in robust linear models.

Statistical significance was evaluated using an unadjusted *p*-value with an alpha threshold of < 0.05. We conducted analysis using R version 4.1.2 [80].

## Supplementary Information

**Additional file 1**. Tables S1–S10 and Figures S1–S2.

## Data Availability

Datasets analyzed in this study are not publicly available; consent for public release of epigenetic data from participants was not obtained from participants. However, data to generate figures and tables are available from the corresponding author with the appropriate permission from the Project Viva study team and investigators (project_viva@hphc.org) upon reasonable request and Institutional Review Board approval. R code for all analyses is available at the study’s GitHub repository (https://github.com/annebozack/ProjectViva_DNAmAge_Predictors).

## Abbreviations

DNAm: DNA methylation
CpG: Cytosine-phosphate-guanine
GA: Gestational age
EA: Epigenetic age
EGA: Epigenetic gestational age
EAA: Epigenetic age acceleration
450K: Illumina HumanMethylation450 array
MoBa: Norwegian mother and child birth cohort
27K: Illumina HumanMethylation27 array
EGAA: Epigenetic gestational age acceleration
BMI: Body mass index
MAE: Median absolute error
NK: Natural killer
nRBCs: Nucleated red blood cells
CI: Confidence interval
ALSPAC: Avon longitudinal study of parents and children
IPA: Ingenuity pathway analysis
IRB: Institutional review board
RBCs: Red blood cells
noob: Normal-exponential out-of-band method
BMIQ: Beta-mixture quantile
LMP: Last menstrual period
