# Supplementary material for: DNA methylation age at birth and childhood: performance of epigenetic clocks and characteristics associated with epigenetic age acceleration in the Project Viva cohort
Source: Clin Epigenetics. 2023 Apr 12;15:62. doi: 10.1186/s13148-023-01480-2 (PMC10099681; doi:10.1186/s13148-023-01480-2)
Supplement: Supplementary file 1 — Additional file 1. Tables S1–S10 and Figures S1–S2. [file 13148_2023_1480_MOESM1_ESM.docx]

**DNA methylation age at birth and childhood: performance of epigenetic clocks and predictors of epigenetic age acceleration in the Project Viva cohort**

**Additional File 1**

**Table S1:** **Characteristics of mother-child pairs with data available at all three timepoints (birth, early childhood, and mid-childhood) (N = 59).**

|  | **n (%)** | | **Missing (n)** | | ***p ^a^*** |
| --- | --- | --- | --- | --- | --- |
| Maternal characteristics |  |  | |  |  |
| Age at enrollment, years, mean (SD) | 31.8 | (5.5) | | 0 | 0.64 |
| Pre-pregnancy BMI, kg/m^2^, mean (SD) | 25.2 | (5.4) | | 0 | 0.51 |
| Pre-pregnancy obesity, n (%) ^c^ | 10 | (16.9%) | | 0 | 0.79 |
| College graduate, n (%) | 41 | (69.5%) | | 0 | 0.74 |
| Annual household income > $70,000, n (%) | 35 | (64.8%) | | 5 | 0.58 |
| Smoking status, n (%) |  |  | | 0 | 0.10 |
| Former smoker | 13 | (22.0%) | | - |  |
| Smoking during pregnancy | 12 | (20.3%) | | - |  |
| Never smoker | 34 | (57.6%) | | - |  |
| Child characteristics |  |  | |  |  |
| Female | 25 | (42.4%) | | 0 | 0.53 |
| Gestational age, weeks, mean (SD) | 39.5 | (1.3) | | 0 | 0.11 |
| Preterm ^b^ | 2 | (3.4%) | | 0 | 1.00 |
| Birth weight for GA z-score, mean (SD) | 0.5 | (0.9) | | 0 | 0.07 |
| Race/ethnicity, n (%) |  |  | | 0 | 0.70 |
| Asian | 4 | (6.8%) | | - |  |
| Black | 7 | (11.9%) | | - |  |
| Hispanic | 2 | (3.4%) | | - |  |
| More than one race or other | 6 | (10.2%) | | - |  |
| White | 40 | (67.8%) | | - |  |
| Age at early childhood sample collection, years, mean (SD) | 3.4 | (0.5) | | 0 | - |
| Age at mid-childhood sample collection, years, mean (SD) | 7.8 | (0.8) | | 0 | - |
| Epigenetic age measures |  |  | |  |  |
| Birth |  |  | |  |  |
| Bohlin EGA, weeks, mean (SD) | 40.0 | (1.0) | | 0 | 0.020 |
| Bohlin EGAA, weeks, mean (SD) | -0.1 | (0.7) | | 0 | 0.10 |
| Knight EGA, weeks, mean (SD) | 38.6 | (1.5) | | 0 | 0.20 |
| Knight EGAA, weeks, mean (SD) | -0.1 | (1.3) | | 0 | 0.41 |
| Horvath EA, years, mean (SD) | 0.1 | (0.2) | | 0 | 0.47 |
| Horvath EAA, years, mean (SD) | 0.0 | (0.2) | | 0 | 0.53 |
| Skin & blood EA, years, mean (SD) | -0.4 | (0.1) | | 0 | 0.85 |
| Skin & blood EAA, years, mean (SD) | 0.0 | (0.1) | | 0 | 0.75 |
| Early childhood ^d^ |  |  | |  |  |
| Horvath EA, years, mean (SD) | 4.2 | (1.0) | | 0 | 0.91 |
| Horvath EAA, years, mean (SD) | 0.0 | (0.8) | | 0 | 0.63 |
| Skin & blood EA, years, mean (SD) | 2.6 | (0.7) | | 0 | 0.74 |
| Skin & blood EAA, years, mean (SD) | 0.0 | (0.5) | | 0 | 0.88 |
| Mid-childhood ^e^ |  |  | |  |  |
| Horvath EA, years, mean (SD) | 8.8 | (1.8) | | 0 | 0.30 |
| Horvath EAA, years, mean (SD) | -0.1 | (1.6) | | 0 | 0.54 |
| Skin & blood EA, years, mean (SD) | 6.2 | (1.1) | | 0 | 0.07 |
| Skin & blood EAA, years, mean (SD) | -0.2 | (0.9) | | 0 | 0.20 |
| a. *P*-value for difference between the group with data available at all three timepoints (birth, early childhood, and mid-childhood) and the group with data available at birth (N = 485, Table 1 in the main text). *P*-values calculated using the Mann–Whitney test for continuous variables and the Chi-squared test or Fisher’s exact test for categorical variables. b. < 37 weeks gestation. c. BMI ≥ 30 kg/m^2^. d. *P*-value for difference between the group with data available at all three timepoints (birth, early childhood, and mid-childhood) and the group with data available at early childhood (N = 120, Table 1 in the main text). e. *P*-value for difference between the group with data available at all three timepoints (birth, early childhood, and mid-childhood) and the group with data available at mid-childhood (N = 460, Table 1 in the main text). | | | | | |

**Figure S1:** Chronological age adjusted for gestation age (GA) and Horvath DNA methylation age in early and mid-childhood. Chronological age was adjusted for GA by adding GA in years to chronological age. The linear trendline and 95% CI is plotted as a solid line and shaded area. The identity line is plotted as a dashed line. r = Pearson’s correlation coefficient; MAE = median absolute error.


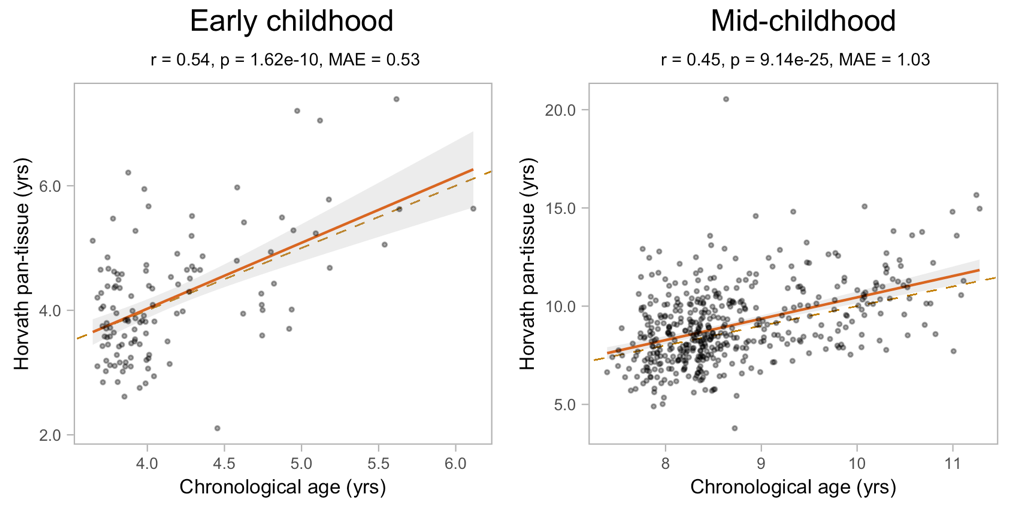


**Table S2: Pearson correlations between** **epigenetic gestational age acceleration (EGAA) and epigenetic age acceleration (EAA) at birth (N = 485) and in mid-childhood (N = 238).**

|  | **EAA at birth** | | | | **Mid-childhood** | | | |
| --- | --- | --- | --- | --- | --- | --- | --- | --- |
|  | **Horvath EAA** | | **Skin & blood EAA** | | **Horvath EAA** | | **Skin & blood EAA** | |
|  | ***r*** | ***p*** | ***r*** | ***p*** | ***r*** | ***p*** | ***r*** | ***p*** |
| **Bohlin EGAA** | 0.17 | <0.001 | 0.13 | 0.005 | 0.04 | 0.51 | 0.03 | 0.59 |
| **Knight EGAA** | 0.19 | <0.001 | 0.21 | <0.001 | 0.04 | 0.57 | 0.08 | 0.23 |

**Table S3: Multivariable associations of maternal-child characteristics with epigenetic gestational age acceleration (EGAA) and epigenetic age acceleration (EAA) at birth, assessed in cord blood (N = 484).** Associations were evaluated using mutually adjusted robust linear regression with and without adjustment for estimated cell type proportions and reported in weeks. Significant associations (*p* < 0.05) are bolded.

|  | **Bohlin EGAA** | | | **Knight EGAA** | | | **Horvath EAA** | | | **Skin & blood EAA** | | |
| --- | --- | --- | --- | --- | --- | --- | --- | --- | --- | --- | --- | --- |
|  | ***B*** | **95% CI** | ***p*** | ***B*** | **95% CI** | ***p*** | ***B*** | **95% CI** | ***p*** | ***B*** | **95% CI** | ***p*** |
| **Adjusted for cell type** |  |  |  |  |  |  |  |  |  |  |  |  |
| Maternal age (per 2 SD change) | -0.03 | (-0.17, 0.11) | 0.70 | 0.08 | (-0.21, 0.38) | 0.58 | -0.69 | (-2.43, 1.05) | 0.44 | -0.08 | (-0.93, 0.77) | 0.86 |
| Pre-pregnancy BMI (per 2 SD change) | 0.07 | (-0.09, 0.22) | 0.39 | -0.14 | (-0.41, 0.13) | 0.30 | -0.79 | (-2.46, 0.88) | 0.36 | -0.16 | (-1.00, 0.69) | 0.71 |
| College graduate (vs. < college graduate) | 0.09 | (-0.08, 0.26) | 0.28 | 0.07 | (-0.20, 0.34) | 0.62 | -1.14 | (-3.11, 0.82) | 0.26 | 0.25 | (-0.64, 1.14) | 0.58 |
| Maternal smoking |  |  |  |  |  |  |  |  |  |  |  |  |
| Former smoker (vs. never) | -0.14 | (-0.31, 0.02) | 0.10 | 0.08 | (-0.19, 0.35) | 0.55 | 0.66 | (-1.29, 2.61) | 0.51 | 0.17 | (-0.79, 1.12) | 0.73 |
| Smoking during pregnancy (vs. never) | -0.12 | (-0.32, 0.08) | 0.23 | 0.24 | (-0.10, 0.58) | 0.17 | -1.24 | (-3.83, 1.34) | 0.35 | 1.17 | (-0.09, 2.42) | 0.07 |
| Female (vs. male) | **-0.17** | **(-0.30, -0.04)** | **0.012** | 0.07 | (-0.16, 0.30) | 0.55 | **-2.88** | **(-4.41, -1.35)** | **<0.001** | -0.55 | (-1.34, 0.24) | 0.18 |
| Preterm (vs. term) | -0.27 | (-0.65, 0.12) | 0.18 | 0.06 | (-0.45, 0.57) | 0.80 | -0.38 | (-4.87, 4.11) | 0.87 | -0.73 | (-2.49, 1.02) | 0.41 |
| Birth weight for GA z-score (per 1 unit) | 0.01 | (-0.06, 0.07) | 0.84 | -0.01 | (-0.13, 0.11) | 0.87 | 0.48 | (-0.31, 1.27) | 0.24 | 0.14 | (-0.29, 0.58) | 0.51 |
| Newborn race/ethnicity ^a^ |  |  |  |  |  |  |  |  |  |  |  |  |
| Asian (vs. White) | -0.31 | (-0.63, 0.00) | 0.05 | **-0.93** | **(-1.66, -0.20)** | **0.013** | -0.16 | (-3.28, 2.96) | 0.92 | -1.22 | (-3.05, 0.61) | 0.19 |
| Black (vs. White) | 0.01 | (-0.22, 0.24) | 0.93 | -0.08 | (-0.46, 0.29) | 0.66 | -0.64 | (-3.19, 1.90) | 0.62 | **1.96** | **(0.62, 3.30)** | **0.004** |
| Hispanic (vs. White) | -0.05 | (-0.31, 0.22) | 0.72 | -0.31 | (-0.89, 0.28) | 0.30 | -0.84 | (-4.32, 2.64) | 0.64 | 1.00 | (-0.93, 2.92) | 0.31 |
| More than one race or other (vs. White) | **0.25** | **(0.01, 0.49)** | **0.038** | -0.02 | (-0.38, 0.33) | 0.89 | -1.86 | (-4.24, 0.52) | 0.13 | 0.49 | (-0.77, 1.76) | 0.45 |
| **Not adjusted for cell type** |  |  |  |  |  |  |  |  |  |  |  |  |
| Maternal age (per 2 SD change) | -0.03 | (-0.17, 0.11) | 0.65 | 0.03 | (-0.28, 0.33) | 0.87 | -0.76 | (-2.60, 1.08) | 0.42 | 0.02 | (-0.96, 0.99) | 0.97 |
| Pre-pregnancy BMI (per 2 SD change) | 0.09 | (-0.06, 0.23) | 0.24 | -0.10 | (-0.37, 0.18) | 0.50 | -1.09 | (-2.93, 0.76) | 0.25 | -0.11 | (-1.10, 0.88) | 0.83 |
| College graduate (vs. < college graduate) | 0.12 | (-0.05, 0.29) | 0.18 | 0.17 | (-0.11, 0.45) | 0.24 | -0.69 | (-2.68, 1.30) | 0.50 | 0.66 | (-0.24, 1.57) | 0.15 |
| Maternal smoking |  |  |  |  |  |  |  |  |  |  |  |  |
| Former smoker (vs. never) | -0.10 | (-0.27, 0.07) | 0.23 | 0.18 | (-0.11, 0.48) | 0.23 | 0.97 | (-1.18, 3.13) | 0.38 | 0.43 | (-0.58, 1.44) | 0.40 |
| Smoking during pregnancy (5vs. never) | -0.12 | (-0.31, 0.07) | 0.22 | 0.24 | (-0.15, 0.63) | 0.23 | -1.10 | (-3.73, 1.54) | 0.41 | **1.87** | **(0.51, 3.23)** | **0.007** |
| Female (vs. male) | -0.11 | (-0.23, 0.02) | 0.09 | **0.27** | **(0.03, 0.51)** | **0.030** | **-2.58** | **(-4.19, -0.97)** | **0.002** | -0.77 | (-1.59, 0.05) | 0.07 |
| Preterm (vs. term) | **-0.42** | **(-0.75, -0.09)** | **0.014** | **-0.54** | **(-1.02, -0.07)** | **0.025** | -0.46 | (-5.25, 4.33) | 0.85 | **-1.94** | **(-3.56, -0.31)** | **0.019** |
| Birth weight for GA z-score (per 1 unit) | 0.01 | (-0.06, 0.08) | 0.74 | 0.00 | (-0.13, 0.13) | 0.97 | 0.13 | (-0.70, 0.97) | 0.75 | 0.01 | (-0.48, 0.49) | 0.98 |
| Newborn race/ethnicity ^a^ |  |  |  |  |  |  |  |  |  |  |  |  |
| Asian (vs. White) | **-0.38** | **(-0.74, -0.02)** | **0.041** | **-1.05** | **(-1.85, -0.25)** | **0.010** | -2.14 | (-5.52, 1.25) | 0.22 | **-2.78** | **(-4.55, -1.02)** | **0.002** |
| Black (vs. White) | -0.03 | (-0.22, 0.17) | 0.80 | -0.35 | (-0.72, 0.02) | 0.07 | -2.63 | (-5.35, 0.09) | 0.06 | **1.88** | **(0.56, 3.20)** | **0.005** |
| Hispanic (vs. White) | 0.00 | (-0.26, 0.25) | 0.98 | -0.19 | (-0.70, 0.31) | 0.45 | -1.49 | (-5.49, 2.52) | 0.47 | 1.61 | (-0.83, 4.05) | 0.20 |
| More than one race or other (vs. White) | 0.24 | (0.00, 0.48) | 0.05 | -0.11 | (-0.52, 0.30) | 0.60 | **-3.46** | **(-5.94, -0.99)** | **0.006** | -0.36 | (-1.58, 0.86) | 0.56 |

**Table S4: Multivariable associations of maternal-child characteristics with epigenetic gestational age acceleration (EGAA) and epigenetic age acceleration (EAA) at birth, assessed in cord blood, among female newborns (N = 254).** Associations were evaluated using mutually adjusted robust linear regression with adjustment for estimated cell type proportions and reported in weeks. Significant associations (*p* < 0.05) are bolded.

|  | **Bohlin EGAA** | | | **Knight EGAA** | | | **Horvath EAA** | | | **Skin & blood EAA** | | |
| --- | --- | --- | --- | --- | --- | --- | --- | --- | --- | --- | --- | --- |
|  | ***B*** | **95% CI** | ***p*** | ***B*** | **95% CI** | ***p*** | ***B*** | **95% CI** | ***p*** | ***B*** | **95% CI** | ***p*** |
| **Adjusted for cell type** |  |  |  |  |  |  |  |  |  |  |  |  |
| Maternal age (per 2 SD change) | -0.02 | (-0.21, 0.17) | 0.82 | -0.21 | (-0.66, 0.23) | 0.35 | -0.73 | (-3.52, 2.07) | 0.61 | -0.23 | (-1.64, 1.19) | 0.75 |
| Pre-pregnancy BMI (per 2 SD change) | **0.20** | **(0.02, 0.37)** | **0.027** | 0.29 | (-0.17, 0.76) | 0.22 | -1.14 | (-4.34, 2.07) | 0.49 | 0.03 | (-1.42, 1.47) | 0.97 |
| College graduate (vs. < college graduate) | 0.10 | (-0.14, 0.34) | 0.43 | 0.18 | (-0.27, 0.63) | 0.43 | -1.41 | (-4.10, 1.27) | 0.30 | 0.24 | (-1.08, 1.55) | 0.73 |
| Maternal smoking |  |  |  |  |  |  |  |  |  |  |  |  |
| Former smoker (vs. never) | **-0.22** | **(-0.44, 0.00)** | **0.045** | -0.07 | (-0.44, 0.30) | 0.70 | -0.48 | (-3.22, 2.26) | 0.73 | -0.25 | (-1.52, 1.02) | 0.70 |
| Smoking during pregnancy (vs. never) | 0.04 | (-0.24, 0.32) | 0.76 | -0.38 | (-1.02, 0.26) | 0.24 | 0.68 | (-3.63, 5.00) | 0.76 | -0.50 | (-2.65, 1.66) | 0.65 |
| Preterm (vs. term) | -0.06 | (-0.65, 0.53) | 0.85 | 0.24 | (-0.45, 0.92) | 0.49 | 1.65 | (-5.02, 8.33) | 0.63 | -0.30 | (-3.26, 2.67) | 0.85 |
| Birth weight for GA z-score (per 1 unit) | 0.00 | (-0.08, 0.08) | 0.94 | -0.07 | (-0.24, 0.10) | 0.40 | -0.30 | (-1.47, 0.86) | 0.61 | 0.04 | (-0.66, 0.73) | 0.92 |
| Newborn race/ethnicity ^a^ |  |  |  |  |  |  |  |  |  |  |  |  |
| Asian (vs. White) | -0.39 | (-0.83, 0.05) | 0.08 | -1.14 | (-2.7, 0.43) | 0.15 | -1.73 | (-7.11, 3.65) | 0.53 | -0.38 | (-3.96, 3.21) | 0.84 |
| Black (vs. White) | 0.00 | (-0.36, 0.36) | 1.00 | -0.32 | (-0.89, 0.26) | 0.29 | -0.68 | (-5.01, 3.65) | 0.76 | 2.14 | (-0.32, 4.60) | 0.09 |
| Hispanic (vs. White) | -0.11 | (-0.47, 0.25) | 0.54 | -0.66 | (-1.59, 0.28) | 0.17 | **-6.13** | **(-10.51, -1.74)** | **0.006** | 2.07 | (-2.40, 6.54) | 0.37 |
| More than one race or other (vs. White) | 0.07 | (-0.21, 0.36) | 0.62 | -0.07 | (-0.50, 0.37) | 0.77 | -1.87 | (-6.10, 2.37) | 0.39 | 0.70 | (-0.71, 2.11) | 0.37 |

**Table S5: Multivariable associations of maternal-child characteristics with epigenetic gestational age acceleration (EGAA) and epigenetic age acceleration (EAA) at birth, assessed in cord blood, among male newborns (N = 230).** Associations were evaluated using mutually adjusted robust linear regression with adjustment for estimated cell type proportions and reported in weeks. Significant associations (*p* < 0.05) are bolded.

|  | **Bohlin EGAA** | | | **Knight EGAA** | | | **Horvath EAA** | | | **Skin & blood EAA** | | |
| --- | --- | --- | --- | --- | --- | --- | --- | --- | --- | --- | --- | --- |
|  | ***B*** | **95% CI** | ***p*** | ***B*** | **95% CI** | ***p*** | ***B*** | **95% CI** | ***p*** | ***B*** | **95% CI** | ***p*** |
| **Adjusted for cell type** |  |  |  |  |  |  |  |  |  |  |  |  |
| Maternal age (per 2 SD change) | -0.04 | (-0.25, 0.17) | 0.72 | 0.30 | (-0.11, 0.70) | 0.15 | -0.77 | (-3.23, 1.70) | 0.54 | 0.03 | (-1.17, 1.22) | 0.97 |
| Pre-pregnancy BMI (per 2 SD change) | -0.12 | (-0.38, 0.14) | 0.37 | **-0.46** | **(-0.83, -0.09)** | **0.014** | -0.27 | (-2.31, 1.78) | 0.80 | -0.32 | (-1.56, 0.91) | 0.61 |
| College graduate (vs. < college graduate) | 0.10 | (-0.16, 0.35) | 0.46 | 0.05 | (-0.35, 0.45) | 0.80 | -0.70 | (-3.64, 2.25) | 0.64 | 0.33 | (-0.92, 1.57) | 0.61 |
| Maternal smoking |  |  |  |  |  |  |  |  |  |  |  |  |
| Former smoker (vs. never) | -0.07 | (-0.31, 0.17) | 0.56 | 0.22 | (-0.18, 0.63) | 0.28 | 1.28 | (-1.60, 4.17) | 0.38 | 0.59 | (-0.98, 2.16) | 0.46 |
| Smoking during pregnancy (vs. never) | -0.12 | (-0.37, 0.14) | 0.37 | **0.67** | **(0.24, 1.10)** | **0.002** | -2.20 | (-5.33, 0.93) | 0.17 | **2.25** | **(0.73, 3.77)** | **0.004** |
| Preterm (vs. term) | -0.33 | (-0.73, 0.08) | 0.11 | -0.07 | (-0.73, 0.60) | 0.85 | -2.73 | (-7.61, 2.15) | 0.27 | -1.51 | (-3.73, 0.71) | 0.18 |
| Birth weight for GA z-score (per 1 unit) | 0.04 | (-0.06, 0.15) | 0.43 | 0.08 | (-0.10, 0.26) | 0.39 | **1.16** | **(0.10, 2.21)** | **0.032** | 0.27 | (-0.32, 0.86) | 0.3**7** |
| Newborn race/ethnicity ^a^ |  |  |  |  |  |  |  |  |  |  |  |  |
| Asian (vs. White) | -0.25 | (-0.72, 0.22) | 0.29 | **-0.67** | **(-1.26, -0.07)** | **0.028** | 0.61 | (-3.40, 4.63) | 0.77 | **-1.89** | **(-3.63, -0.15)** | **0.033** |
| Black (vs. White) | 0.00 | (-0.32, 0.33) | 1.00 | 0.09 | (-0.46, 0.64) | 0.76 | 0.09 | (-3.04, 3.22) | 0.95 | **2.10** | **(0.41, 3.78)** | **0.015** |
| Hispanic (vs. White) | 0.07 | (-0.32, 0.45) | 0.73 | 0.06 | (-0.79, 0.91) | 0.89 | 2.34 | (-1.56, 6.24) | 0.24 | 0.94 | (-1.60, 3.47) | 0.47 |
| More than one race or other (vs. White) | **0.49** | **(0.17, 0.80)** | **0.003** | 0.14 | (-0.39, 0.66) | 0.61 | -2.00 | (-4.97, 0.98) | 0.19 | 0.52 | (-1.54, 2.58) | 0.62 |

**Table S6: Multivariable associations of maternal-child characteristics with epigenetic age acceleration (EAA) in early (N = 119) and mid-childhood (N = 455).** Associations were evaluated using mutually adjusted robust linear regression with and without adjustment for estimated cell type proportions and reported in years. Significant associations (*p* < 0.05) are bolded.

|  | **Early childhood** | | | | | | **Mid-childhood** | | | | | |
| --- | --- | --- | --- | --- | --- | --- | --- | --- | --- | --- | --- | --- |
|  | **Horvath EAA** | | | **Skin & blood EAA** | | | **Horvath EAA** | | | **Skin & blood EAA** | | |
|  | ***B*** | **95% CI** | ***p*** | ***B*** | **95% CI** | ***p*** | ***B*** | **95% CI** | ***p*** | ***B*** | **95% CI** | ***p*** |
| **Adjusted for cell type** |  |  |  |  |  |  |  |  |  |  |  |  |
| Maternal age (per 2 SD change) | -0.06 | (-0.47, 0.34) | 0.75 | -0.01 | (-0.24, 0.23) | 0.96 | -0.31 | (-0.64, 0.01) | 0.06 | -0.05 | (-0.25, 0.16) | 0.67 |
| Pre-pregnancy BMI (per 2 SD change) | -0.06 | (-0.39, 0.26) | 0.69 | -0.13 | (-0.34, 0.07) | 0.20 | -0.19 | (-0.57, 0.20) | 0.34 | 0.18 | (-0.01, 0.36) | 0.07 |
| College graduate (vs. < college graduate) | **0.34** | **(0.04, 0.63)** | **0.028** | -0.03 | (-0.28, 0.23) | 0.84 | -0.16 | (-0.52, 0.20) | 0.38 | -0.08 | (-0.31, 0.15) | 0.49 |
| Maternal smoking |  |  |  |  |  |  |  |  |  |  |  |  |
| Former smoker (vs. never) | 0.02 | (-0.38, 0.42) | 0.92 | -0.04 | (-0.24, 0.17) | 0.73 | -0.25 | (-0.66, 0.16) | 0.23 | -0.06 | (-0.29, 0.18) | 0.64 |
| Smoking during pregnancy (vs. never) | 0.45 | (-0.09, 0.99) | 0.10 | **0.34** | **(0.03, 0.64)** | **0.032** | -0.22 | (-0.72, 0.29) | 0.40 | 0.02 | (-0.26, 0.30) | 0.90 |
| Female (vs. male) | -0.30 | (-0.60, 0.01) | 0.06 | 0.02 | (-0.17, 0.22) | 0.80 | **-0.48** | **(-0.77, -0.18)** | **0.002** | 0.13 | (-0.05, 0.31) | 0.15 |
| Preterm (vs. term) | -0.54 | (-1.24, 0.17) | 0.14 | -0.01 | (-0.41, 0.40) | 0.97 | -0.23 | (-0.77, 0.30) | 0.40 | **-0.62** | **(-0.96, -0.28)** | **<0.001** |
| Birth weight for GA z-score (per 1 unit) | -0.08 | (-0.24, 0.08) | 0.35 | -0.03 | (-0.13, 0.07) | 0.53 | 0.06 | (-0.13, 0.25) | 0.54 | 0.04 | (-0.06, 0.15) | 0.42 |
| Child race/ethnicity ^a^ |  |  |  |  |  |  |  |  |  |  |  |  |
| Asian (vs. White) | -0.31 | (-0.80, 0.18) | 0.22 | -0.09 | (-0.50, 0.32) | 0.68 | 0.76 | (-0.14, 1.66) | 0.10 | 0.17 | (-0.22, 0.56) | 0.40 |
| Black (vs. White) | -0.03 | (-0.59, 0.54) | 0.93 | 0.02 | (-0.70, 0.75) | 0.95 | -0.22 | (-0.72, 0.27) | 0.38 | 0.20 | (-0.11, 0.51) | 0.21 |
| Hispanic (vs. White) | -0.14 | (-0.74, 0.45) | 0.64 | 0.26 | (-0.25, 0.77) | 0.32 | 0.51 | (-0.20, 1.23) | 0.16 | **0.83** | **(0.37, 1.30)** | **<0.001** |
| More than one race or other (vs. White) | -0.02 | (-0.67, 0.63) | 0.95 | 0.35 | (-0.04, 0.75) | 0.08 | 0.02 | (-0.43, 0.48) | 0.92 | 0.18 | (-0.10, 0.45) | 0.21 |
| **Not adjusted for cell type** |  |  |  |  |  |  |  |  |  |  |  |  |
| Maternal age (per 2 SD change) | -0.09 | (-0.46, 0.28) | 0.64 | -0.01 | (-0.26, 0.23) | 0.91 | **-0.35** | **(-0.69, 0.00)** | **0.048** | -0.05 | (-0.26, 0.15) | 0.60 |
| Pre-pregnancy BMI (per 2 SD change) | -0.05 | (-0.38, 0.27) | 0.74 | -0.07 | (-0.28, 0.13) | 0.49 | -0.23 | (-0.60, 0.15) | 0.23 | 0.19 | (-0.02, 0.39) | 0.07 |
| College graduate (vs. < college graduate) | 0.31 | (-0.03, 0.65) | 0.07 | -0.02 | (-0.23, 0.18) | 0.83 | -0.18 | (-0.56, 0.20) | 0.35 | -0.11 | (-0.37, 0.15) | 0.39 |
| Maternal smoking |  |  |  |  |  |  |  |  |  |  |  |  |
| Former smoker (vs. never) | 0.06 | (-0.33, 0.45) | 0.77 | -0.02 | (-0.24, 0.20) | 0.85 | -0.20 | (-0.59, 0.19) | 0.31 | -0.01 | (-0.26, 0.23) | 0.91 |
| Smoking during pregnancy (vs. never) | 0.34 | (-0.12, 0.80) | 0.15 | 0.26 | (-0.01, 0.54) | 0.06 | -0.31 | (-0.83, 0.21) | 0.24 | 0.04 | (-0.26, 0.35) | 0.78 |
| Female (vs. male) | -0.15 | (-0.44, 0.13) | 0.29 | 0.03 | (-0.16, 0.23) | 0.74 | **-0.54** | **(-0.85, -0.23)** | **0.001** | 0.11 | (-0.08, 0.30) | 0.24 |
| Preterm (vs. term) | -0.27 | (-0.89, 0.34) | 0.38 | 0.13 | (-0.32, 0.57) | 0.57 | -0.23 | (-0.82, 0.36) | 0.45 | **-0.71** | **(-1.06, -0.37)** | **<0.001** |
| Birth weight for GA z-score (per 1 unit) | 0.01 | (-0.16, 0.18) | 0.91 | -0.04 | (-0.16, 0.07) | 0.46 | 0.03 | (-0.17, 0.23) | 0.76 | 0.03 | (-0.09, 0.14) | 0.66 |
| Child race/ethnicity ^a^ |  |  |  |  |  |  |  |  |  |  |  |  |
| Asian (vs. White) | 0.10 | (-0.35, 0.54) | 0.67 | 0.08 | (-0.37, 0.52) | 0.73 | 0.82 | (-0.20, 1.84) | 0.11 | 0.09 | (-0.32, 0.49) | 0.67 |
| Black (vs. White) | 0.27 | (-0.36, 0.90) | 0.40 | 0.05 | (-0.60, 0.70) | 0.88 | -0.24 | (-0.72, 0.24) | 0.32 | 0.04 | (-0.30, 0.37) | 0.83 |
| Hispanic (vs. White) | 0.20 | (-0.45, 0.86) | 0.54 | 0.23 | (-0.75, 1.22) | 0.65 | 0.59 | (-0.16, 1.34) | 0.12 | **0.71** | **(0.25, 1.18)** | **0.003** |
| More than one race or other (vs. White) | 0.08 | (-0.45, 0.62) | 0.76 | 0.33 | (-0.08, 0.74) | 0.11 | 0.02 | (-0.48, 0.52) | 0.94 | 0.08 | (-0.23, 0.38) | 0.62 |

**Table S7: Multivariable associations of maternal-child characteristics with epigenetic age acceleration (EAA) in early (N = 58) and mid-childhood among female children (N = 218).** Associations were evaluated using mutually adjusted robust linear regression with and without adjustment for estimated cell type proportions and reported in years. Significant associations (*p* < 0.05) are bolded.

|  | **Early childhood** | | | | | | **Mid-childhood** | | | | | |
| --- | --- | --- | --- | --- | --- | --- | --- | --- | --- | --- | --- | --- |
|  | **Horvath EAA** | | | **Skin & blood EAA** | | | **Horvath EAA** | | | **Skin & blood EAA** | | |
|  | ***B*** | **95% CI** | ***p*** | ***B*** | **95% CI** | ***p*** | ***B*** | **95% CI** | ***p*** | ***B*** | **95% CI** | ***p*** |
| **Adjusted for cell type** |  |  |  |  |  |  |  |  |  |  |  |  |
| Maternal age (per 2 SD change) | 0.24 | (-0.44, 0.93) | 0.48 | 0.09 | (-0.21, 0.39) | 0.56 | 0.17 | (-0.26, 0.59) | 0.44 | 0.26 | (-0.08, 0.60) | 0.13 |
| Pre-pregnancy BMI (per 2 SD change) | -0.09 | (-0.43, 0.24) | 0.60 | -0.10 | (-0.30, 0.10) | 0.34 | -0.25 | (-0.80, 0.29) | 0.36 | 0.28 | (-0.02, 0.57) | 0.06 |
| College graduate (vs. < college graduate) | 0.20 | (-0.53, 0.93) | 0.59 | -0.36 | (-0.80, 0.08) | 0.11 | -0.27 | (-0.91, 0.36) | 0.40 | -0.33 | (-0.71, 0.04) | 0.08 |
| Maternal smoking |  |  |  |  |  |  |  |  |  |  |  |  |
| Former smoker (vs. never) | 0.08 | (-0.53, 0.68) | 0.80 | -0.16 | (-0.45, 0.12) | 0.27 | -0.19 | (-0.77, 0.39) | 0.52 | 0.01 | (-0.36, 0.38) | 0.95 |
| Smoking during pregnancy (vs. never) | 0.40 | (-0.17, 0.97) | 0.17 | **0.53** | **(0.17, 0.88)** | **0.004** | -0.06 | (-0.92, 0.80) | 0.88 | 0.08 | (-0.40, 0.56) | 0.75 |
| Preterm (vs. term) | **-0.59** | **(-1.14, -0.04)** | **0.035** | 0.20 | (-0.03, 0.44) | 0.09 | 0.23 | (-0.61, 1.06) | 0.60 | **-0.89** | **(-1.46, -0.33)** | **0.002** |
| Birth weight for GA z-score (per 1 unit) | 0.07 | (-0.21, 0.35) | 0.62 | 0.07 | (-0.08, 0.22) | 0.37 | 0.01 | (-0.27, 0.29) | 0.94 | 0.10 | (-0.09, 0.28) | 0.30 |
| Newborn race/ethnicity ^a^ |  |  |  |  |  |  |  |  |  |  |  |  |
| Asian (vs. White) | -0.02 | (-1.03, 0.99) | 0.96 | -0.02 | (-0.48, 0.43) | 0.92 | 0.30 | (-0.45, 1.04) | 0.43 | -0.10 | (-0.80, 0.61) | 0.79 |
| Black (vs. White) | -0.17 | (-1.24, 0.91) | 0.76 | -0.38 | (-1.38, 0.61) | 0.45 | -0.12 | (-0.83, 0.58) | 0.73 | 0.19 | (-0.33, 0.71) | 0.48 |
| Hispanic (vs. White) | 0.39 | (-0.38, 1.16) | 0.32 | 0.08 | (-0.60, 0.76) | 0.82 | 0.34 | (-0.51, 1.20) | 0.43 | **0.68** | **(0.08, 1.27)** | **0.025** |
| More than one race or other (vs. White) | **0.65** | **(0.15, 1.14)** | **0.010** | **0.49** | **(0.08, 0.90)** | **0.020** | -0.11 | (-0.80, 0.58) | 0.76 | 0.19 | (-0.21, 0.60) | 0.35 |

**Table S8: Multivariable associations of maternal-child characteristics with epigenetic age acceleration (EAA) in early (N = 61) and mid-childhood among male children (N = 237).** Associations were evaluated using mutually adjusted robust linear regression with and without adjustment for estimated cell type proportions and reported in years. Significant associations (*p* < 0.05) are bolded.

|  | **Early childhood** | | | | | | **Mid-childhood** | | | | | |
| --- | --- | --- | --- | --- | --- | --- | --- | --- | --- | --- | --- | --- |
|  | **Horvath EAA** | | | **Skin & blood EAA** | | | **Horvath EAA** | | | **Skin & blood EAA** | | |
|  | ***B*** | **95% CI** | ***p*** | ***B*** | **95% CI** | ***p*** | ***B*** | **95% CI** | ***p*** | ***B*** | **95% CI** | ***p*** |
| **Adjusted for cell type** |  |  |  |  |  |  |  |  |  |  |  |  |
| Maternal age (per 2 SD change) | -0.34 | (-0.87, 0.19) | 0.21 | -0.11 | (-0.48, 0.27) | 0.58 | **-0.83** | **(-1.29, -0.36)** | **0.001** | **-0.29** | **(-0.53, -0.05)** | **0.017** |
| Pre-pregnancy BMI (per 2 SD change) | -0.18 | (-0.60, 0.25) | 0.42 | -0.20 | (-0.64, 0.23) | 0.36 | -0.09 | (-0.64, 0.45) | 0.74 | 0.11 | (-0.12, 0.35) | 0.34 |
| College graduate (vs. < college graduate) | 0.04 | (-0.36, 0.44) | 0.84 | 0.09 | (-0.15, 0.32) | 0.48 | -0.10 | (-0.56, 0.35) | 0.65 | 0.08 | (-0.20, 0.36) | 0.57 |
| Maternal smoking |  |  |  |  |  |  |  |  |  |  |  |  |
| Former smoker (vs. never) | -0.09 | (-0.57, 0.38) | 0.71 | -0.08 | (-0.34, 0.18) | 0.57 | -0.15 | (-0.79, 0.49) | 0.66 | -0.09 | (-0.40, 0.21) | 0.55 |
| Smoking during pregnancy (vs. never) | 0.47 | (-0.40, 1.34) | 0.29 | 0.24 | (-0.50, 0.98) | 0.53 | -0.38 | (-1.04, 0.28) | 0.26 | -0.06 | (-0.41, 0.29) | 0.75 |
| Preterm (vs. term) | -0.57 | (-1.45, 0.31) | 0.21 | -0.19 | (-1.23, 0.85) | 0.72 | **-0.63** | **(-1.25, -0.01)** | **0.047** | **-0.44** | **(-0.82, -0.05)** | **0.026** |
| Birth weight for GA z-score (per 1 unit) | -0.02 | (-0.25, 0.20) | 0.83 | -0.01 | (-0.17, 0.15) | 0.88 | 0.06 | (-0.19, 0.31) | 0.64 | 0.00 | (-0.13, 0.14) | 0.96 |
| Newborn race/ethnicity ^a^ |  |  |  |  |  |  |  |  |  |  |  |  |
| Asian (vs. White) | **-0.59** | **(-1.05, -0.12)** | **0.013** | -0.23 | (-1.37, 0.90) | 0.69 | 1.09 | (-0.64, 2.83) | 0.22 | **0.35** | **(0.02, 0.69)** | **0.037** |
| Black (vs. White) | -0.53 | (-1.18, 0.11) | 0.11 | 0.12 | (-0.26, 0.49) | 0.54 | -0.31 | (-1.00, 0.37) | 0.37 | 0.16 | (-0.21, 0.53) | 0.40 |
| Hispanic (vs. White) | **-1.31** | **(-1.98, -0.64)** | **<0.001** | 0.19 | (-0.32, 0.70) | 0.47 | 0.69 | (-0.72, 2.10) | 0.34 | **0.79** | **(0.14, 1.45)** | **0.018** |
| More than one race or other (vs. White) | -0.01 | (-0.99, 0.97) | 0.99 | 0.62 | (-0.30, 1.55) | 0.19 | 0.14 | (-0.40, 0.68) | 0.61 | 0.17 | (-0.20, 0.53) | 0.36 |

**Table S9: Associations between Horvath epigenetic age (EA, in weeks) in cord blood and epigenetic age acceleration (EAA, in years) in early and mid-childhood.** Associations were evaluated using robust linear regression. Adjusted models included child sex, preterm status, birth weight for gestational age z-score, child race/ethnicity, maternal age, pre-pregnancy BMI, maternal education (college graduate vs. not), maternal smoking (former smoker or smoking during pregnancy vs. never smoker), and estimated cell type proportions.

|  |  | **Early childhood** | | | | | **Mid childhood** | | | | | |
| --- | --- | --- | --- | --- | --- | --- | --- | --- | --- | --- | --- | --- |
|  | **Horvath EAA (years)** | | | **Skin & blood EAA (years)** | | | **Horvath EAA (years)** | | | **Skin & blood EAA (years)** | | |
|  | ***B*** | **95% CI** | ***p*** | ***B*** | **95% CI** | ***p*** | ***B*** | **95% CI** | ***p*** | ***B*** | **95% CI** | ***p*** |
| Cord blood Horvath EA (weeks), unadjusted ^a^ | 0.03 | (0.02, 0.05) | <0.001 | 0.02 | (0.01, 0.03) | <0.001 | 0.04 | (0.02, 0.07) | <0.001 | 0.01 | (0.00, 0.03) | 0.023 |
| Cord blood Horvath EA (weeks), adjusted ^b^ | 0.03 | (0.02, 0.05) | <0.001 | 0.02 | (0.02, 0.03) | <0.001 | 0.05 | (0.03, 0.07) | <0.001 | 0.02 | (0.01, 0.03) | 0.002 |
| a. Early childhood N = 113; mid-childhood N = 238. b. Early childhood N = 112; mid-childhood N = 238. | | | | | | | | | | | | |

**Table S10: Summary of DNA methylation aging biomarkers computed in the study.**

| **Clock** | **Prediction** | **# of probes** | **Tissues used for training** |
| --- | --- | --- | --- |
| Bohlin [1] | Gestational age | 96 or 251 based on penalty | Cord blood (MoBa Cohort) |
| Knight [2] | Gestational age | 148 | Cord blood or blood spots (6 cohorts) |
| Horvath [3] | Pan-tissue chronological age | 353 | 82 datasets with 51 tissues and cell types (adult blood and tissues, cord blood, placenta, and sperm) |
| Skin & blood [4] | Multi-tissue chronological age | 391 | 10 datasets (buccal cells, epithelium, fibroblasts, whole blood, skin, and cord blood) |

**Figure S2: Upset plot of number of CpGs overlapping between clocks.**


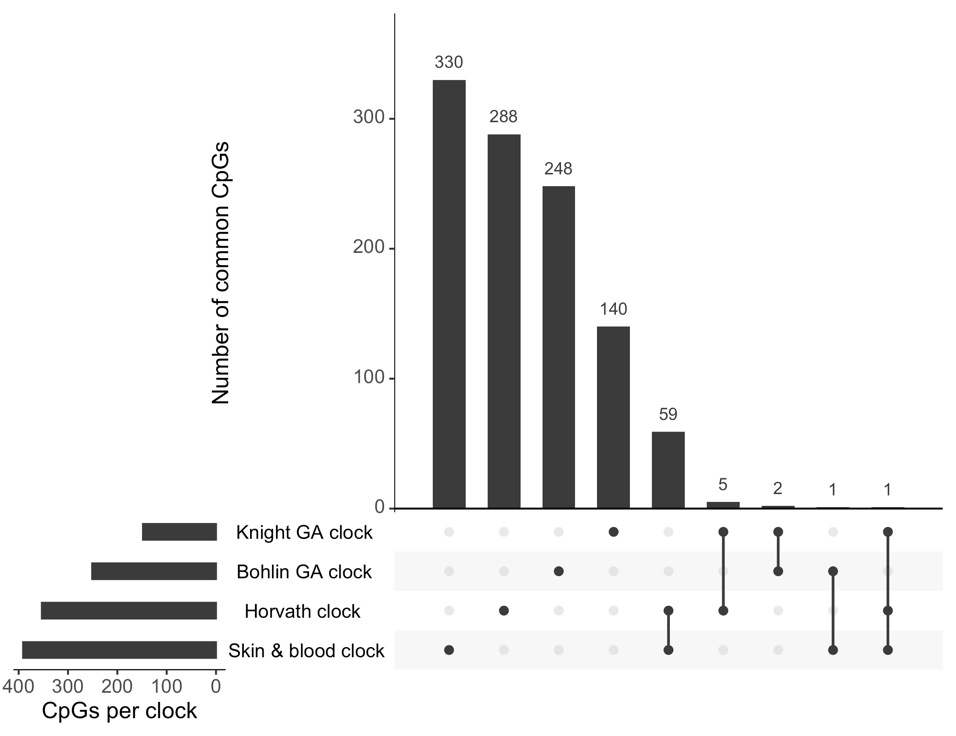


**References**

1. Bohlin J, Håberg SE, Magnus P, Reese SE, Gjessing HK, Magnus MC, et al. Prediction of gestational age based on genome-wide differentially methylated regions. Genome Biol. BioMed Central; 2016;17:207.

2. Knight AK, Craig JM, Theda C, Bækvad-Hansen M, Bybjerg-Grauholm J, Hansen CS, et al. An epigenetic clock for gestational age at birth based on blood methylation data. Genome Biol. 2016;17:206.

3. Horvath S. DNA methylation age of human tissues and cell types. Genome Biol. 2013;14:R115.

4. Horvath S, Oshima J, Martin GM, Lu AT, Quach A, Cohen H, et al. Epigenetic clock for skin and blood cells applied to Hutchinson Gilford Progeria Syndrome and ex vivo studies. Aging. 2018;10:1758–75.
